# Dynamics-informed multigraph neural networks for protein thermostability prediction and residue-level interpretation

**DOI:** 10.1016/j.isci.2026.116089

**Published:** 2026-05-25

**Authors:** Yen-Lin Chen, Shu-Wei Chang

**Affiliations:** 1 Department of Civil Engineering, National Taiwan University, Taipei 106, Taiwan; 2 Department of Biomedical Engineering, National Taiwan University, Taipei 106, Taiwan

**Keywords:** biochemistry, biophysics, protein physics

## Abstract

Thermostability is crucial for protein engineering, but experimental determination of melting temperatures is costly and time-consuming. Most existing machine learning approaches for melting temperature prediction rely on sequential and structural features while largely overlooking protein dynamics, a key determinant of conformational stability. Here, we introduce three dynamics-informed graphs and evaluate their utility relative to sequential or structural representations. Specifically, we propose three dynamical graphs derived from normal mode analysis—co-directionality, coordination, and deformation graphs—and assess whether they can serve as alternatives to contact graphs in predicting melting temperatures. We then integrate sequential, structural, and dynamical information within a unified multigraph learning framework. Our results show that dynamical graphs achieve comparable predictive performance to conventional contact graphs, and that combining structural and dynamical graphs yields consistent, albeit modest, improvements over contact-only models. Furthermore, Laplacian centrality analysis on coordination graphs reveals enrichment tendencies and mechanical signals, providing interpretability. Overall, this work demonstrates the value of protein dynamics-informed multigraph representations for learning protein properties.

## Highlights

• Three novel graphs to encode dynamics: co-directionality, coordination, deformation
• Dynamics-informed graphs match contact graphs in melting temperature prediction
• Multigraph model improves RMSE by 0.07 over contact-only baseline
• Laplacian centrality reveals enrichment tendencies and mechanical signals

## Graphical abstract

**Figure:**
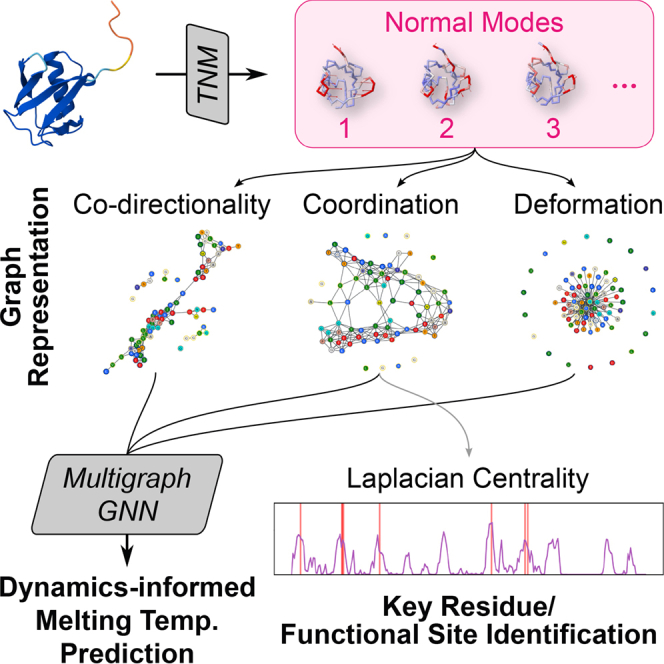

## Introduction

Proteins are nanometric machines essential for the proper operation of organisms, refined by billions of years of evolution and fine-tuned to suit the specific needs of the cellular environment. When removed from living organisms, proteins also find use as enzymes or markers in industrial settings, making protein science a critical field of research both in terms of healthcare and industry. Protein functionality relies on their conformation and protein dynamics,^1^^,^^2^ which are modulated by a variety of conditions, such as membrane tension,^3^ ligand binding,^2^^,^^4^ and temperature. Thermostability is especially critical for proteins that find use outside living organisms, where they are commonly subjected to non-physiological temperatures to either accelerate the process or meet the requirements of other chemicals involved in the process. For example, thermostable polymerases are preferred to accelerate the amplification of the quantity of DNA in a polymerase chain reaction (PCR), and thermostability is critical for PET plastic-degrading enzymes since they are often subjected to unstable environments involving multiple heat treatments.^5^^,^^6^

The thermostability of a protein is commonly characterized by its melting temperature, the temperature at which the folded and unfolded states of the protein exist in equal amounts, which can be measured by techniques like differential scanning calorimetry (DSC)^7^ or circular dichroism (CD) spectroscopy.^8^ However, experimental measures are costly both in terms of time and money, and some effort has been put into the development of computational alternatives taking advantage of recent advances in machine learning (ML). Two models were developed before the publication of large datasets: Gorania et al. proposed a 3-layer artificial neural network in 2010 based on amino acid compositions,^9^ and Yang et al. proposed ProTstab,^10^ a LightGBM model that takes 2,077 sequential and structural features as input, in 2019. The datasets ProThermDB^11^ and Meltome Atlas^12^ were both published in 2020, both including more than 30,000 entries, allowing for the development of larger and deeper ML models. Yang et al. proposed ProTstab2,^13^ another GBM-based model that takes over 6,000 sequential and structural descriptors as input. Jung et al. proposed a multilayer perceptron (MLP) model named DeepSTABp^14^ that takes three features as input: the sequential embedding of ProtTrans,^15^ the type of experimental measurement (cell or lysate), and the optimal growth temperature of the host species. Li et al. proposed DeepTM,^16^ a graph neural network (GNN) developed on contact edges and a conglomerate of node features, including physicochemical properties and evolutionary information from BLOSUM62 and a hidden Markov model (HMM). Rodella et al. proposed TemBERTure,^17^ a framework for predicting thermostability and melting temperature based on sequences. Rollins et al. proposed AbMelt,^18^ a collection of traditional ML models trained on physicochemical descriptors such as radius of gyration, hydrogen bonding, solvent-accessible surface area (SASA), and residue-level root-mean-square fluctuations (RMSF) derived from molecular dynamics (MD) simulations. Tijare et al. proposed PPTStab,^19^ a collection of ML models based on protein sequence embeddings. While most existing predictors rely primarily on sequential and structural features, AbMelt is among the few that incorporate MD-derived information. However, the dynamic information in AbMelt is encoded through aggregated, high-level descriptors computed from explicit MD trajectories, which are computationally expensive to obtain and largely summarize fluctuations around sampled conformations rather than the intrinsic collective motions of the protein. General information on protein dynamics, a property known to be critical for conformational changes and protein functionality, is rarely included in these works, possibly due to challenges in its characterization and data representation.

GNNs have gained considerable traction in recent years in protein science due to the rotational invariance that is intrinsic to graph representations of proteins. These methods cover graph-level prediction of a range of protein properties and behaviors, such as function,^20^^,^^21^^,^^22^^,^^23^ Gibbs free energy change upon mutation,^24^^,^^25^^,^^26^ melting temperature,^16^ natural frequency,^27^ and solubility.^28^^,^^29^^,^^30^ The typical representation has residues as nodes and the contact map as graph edges to encode structural information. Common node features include amino acid type, physicochemical descriptors, evolutionary information like BLOSUM or HMM, secondary structure, and amino acid and dipeptide frequency. On the other hand, common edge features include inter-residue distances and orientation.^31^ These representations cover sequential and structural aspects of the protein to different extents, and protein dynamics is left out of the encoding. Our previous work is among the first to encode protein dynamics into graph representations of proteins.^21^ It was shown that it is possible to enhance the predictability of protein function using a dynamics-informed representation. Furthermore, existing GNN-based predictors rely on simple graphs for protein representation, leaving the potential of multigraph representations and aggregations untapped. A handful of graph aggregation methods that exploit the architecture of multigraphs have been proposed in the literature, reporting improved predictability or expressive power,^32^^,^^33^^,^^34^ but they are not yet widely applied in protein science. Hence, protein dynamics and multigraph-based protein predictors are worthy of further exploration.

Summing up, existing models overlook dynamics and rely almost exclusively on sequential and structural representations. We believe ML models stand to gain much more information if features on general protein dynamics could be established. To address this gap, this work introduces three dynamics-informed graph representations and evaluates their utility for protein thermostability prediction. We further show that dynamical representations are viable replacements for contact graphs, and that the combination of sequential, structural, and dynamical representations provides the best performance when paired with a multigraph-based predictor. Lastly, we analyze the Laplacian centrality of the proposed dynamical graphs to assess their association with residue-level functional annotations, thereby enhancing the interpretability of the proposed graphs.

## Results

### Dynamical graphs provide multifaceted information drastically different from contact graphs

**Figure 1:**
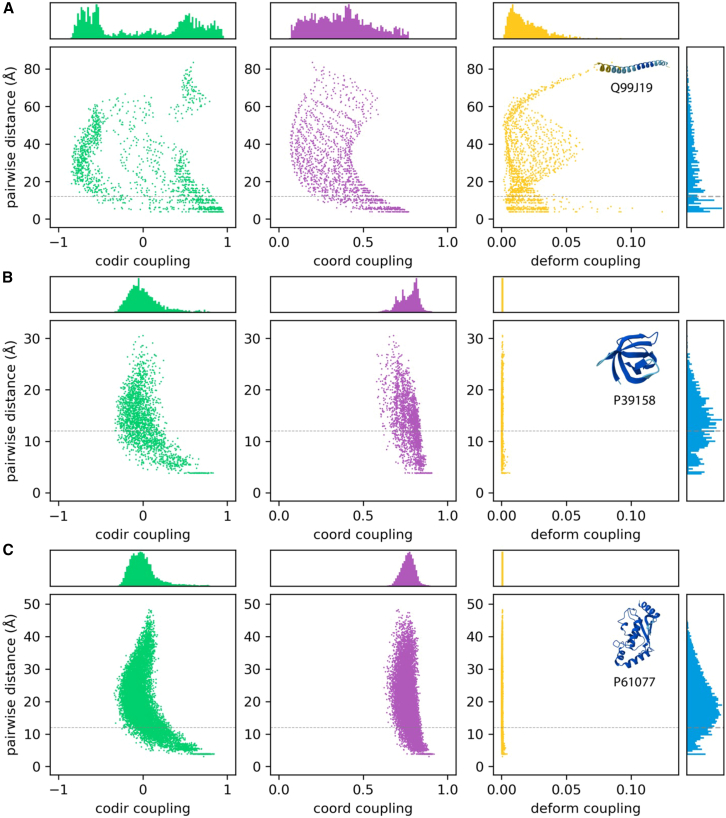
Scatter plot of the three coupling types versus pairwise distance Correlation and comparison between pairwise distance and the three coupling types for (A) helices (UniProt: Q99J19), (B) beta barrels (UniProt: P39158), and (C) a mixture of helices, sheets, and loops (E2 enzyme, UniProt: P61077). The distributions of couplings between the three coupling types are significantly different. Also see Figures S1–S10.

We plotted the correlation between the pairwise dynamical couplings against the pairwise distances, as well as the density histogram of the couplings and distances, to look into the distribution and characteristics of dynamical couplings on individual proteins. The results for a helical protein, a beta barrel, and a typical globular protein consisting of a mixture of helices, strands, and loops are shown in Figure 1.

As shown in Figure 1A, the distribution of the three dynamical couplings is vastly different from each other. While the co-directionality coupling has a bimodal distribution, the distribution is closer to unimodal for coordination and deformation couplings. This is in contrast to the distribution of distances, where a near-linear decrease is observed. The scatterplot revealed another picture of the coupling distributions. In particular, there is an isolated cluster in the scatterplot with a large distance and high co-directionality. By definition, this indicates that residue pairs located furthest apart tend to move in similar directions, and that this movement is mostly discontinuous and unique to the two ends. There is no clear clustering for coordination or deformation coupling. However, it is noteworthy that there seems to be a manifold in a higher dimension when plotting deformation coupling against distance, which could be worthy of further investigation. In contrast to the helical protein, the distributions of all three dynamical couplings are tightly packed for the beta barrel and the mixed structure shown in Figures 1B and 1C. Both the co-directionality and coordination coupling showed a unimodal, bell-shaped distribution for these proteins. The scatterplot revealed that co-directionality is dependent on the pairwise distance for both proteins, with higher coupling between residues that are either close or far apart, but lower coupling for moderately spaced pairs. On the other hand, coordination is less sensitive to distance for both proteins; this is especially so for the protein with a mixed structure.

**Figure 2:**
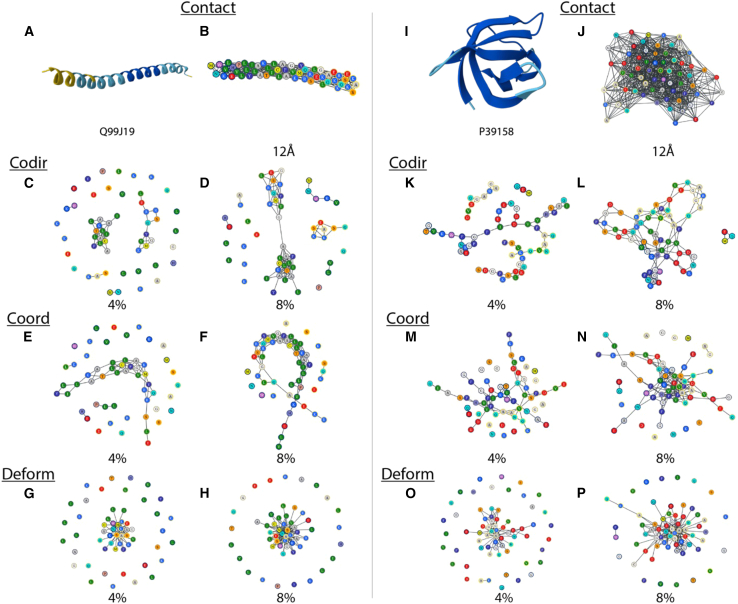
The three different types of dynamical edge connections for a helical protein (UniProt: Q99J19) and a beta barrel (UniProt: P39158) (A and J) Snapshot of the structure from AlphaFoldDB in ribbon representation. (B and K) The contact edges with 12 Å as cutoff. Graph visualization with the scalable force-directed placement (SFDP) layout is shown for (C, D, L, and M) co-directionality, (E, F, N, and O) coordination, and (G, H, P, and Q) deformation edges. Also see Figures S1–S10.

We also visualized the three dynamical edge types using gradually increasing thresholds to understand the effect of thresholding on the representation. This is shown for the helical protein and beta barrel in Figure 2, where the scalable force-directed placement (SFDP) method is used for the node layout. The percentages are computed with respect to the total possible number of connections for each protein, e.g., 4% corresponds to the threshold being selected such that there will be approximately [n×(n−1)/2]×4% edges after thresholding for a protein with n residues. More proteins are shown in Figures S1–S10.

First and foremost, the three different types of dynamical couplings resulted in vastly different graph structures. For the helix, co-directionality graphs tend to form clusters that are gradually connected as the threshold is decreased and more edges are introduced. Hence, the coupling tends to connect residues within the same subgraph before reaching out to residues outside the cluster. A major structure forms in the coordination graph early on that only changes gradually as more edges are introduced. This is likely due to the edges being distributed evenly among connected residues. Deformation graphs demonstrated a centralized structure that gradually grows larger as the threshold is increased.

In comparison to the co-directionality graph of the helical protein, the co-directionality graph of the beta barrel rapidly connects the majority of residues in the protein. This is possibly because the barrel is a structure more tightly packed than helices, where every residue pair is closer and therefore has more impact on each other. The co-directionality edges are distributed more evenly as a result. The coordination graph again develops a major structure that remains relatively unchanged as more edges are introduced, similar to that of the helical structure. The behavior of the deformation graph is also similar to the previous cases, where a centralized cluster is obvious.

Overall, the major structure of the coordination graph emerges early on. This implies that a larger threshold can be used, and a lower number of edges are required to characterize the protein. On the other hand, deformation graphs have centralized structures in both cases. By definition, this subset would be a group of residues that are sensitive to forces applied to each other. It would be a good point of reference when looking into catalytic and allosteric sites. There are no clear trends for co-directionality graphs, as the structure seems to depend heavily on characteristics of the protein itself.

### Dynamical graphs are viable substitutes for contact graphs in melting temperature prediction

We compare the effectiveness of the four edge types using the S1 feature extractor. The cutoff for the contact graph is fixed at 12 Å. Of all the thresholds tested for the three dynamical graphs (as listed in Tables 1 and S1) and excluding graphs with less than 10 edges on average per protein on the preliminary set, the best-performing definition is 20 N for co-directionality graphs, 1DCONT for coordination graphs, and 2DSIGMA for deformation graphs.

**Table 1:** List of threshold definitions tested

	Codename	Definition	Values Tested
Entry-by-entry	N	for each protein, the threshold is set to the value at (100−N)% of the min-max coupling range	5, 10, 20
	SIGMA	for each protein, select edges with couplings larger than μ+(SIGMA)×σ	1.5, 2, 3
	CONT	for each protein, select edges with coupling that rank within the top $\left(\text{CONT}\right)\times N_{\text{cont}}$	0.5, 1, 1.5
	PAIR	for each protein, select edges with coupling that rank within the top (PAIR)%×L×(L−1)/2	10, 20, 30
Dataset-wide	DN	for the entire set of proteins, the threshold is set to the value at (100−DN)% of the min–max coupling range of the training set	10, 20, 30
	DSIGMA	for the entire set of proteins, select the edges with couplings larger than μ+(DSIGMA)×σ, where μ and σ are computed w.r.t. all proteins in the training set	1.5, 2, 3
	DCONT	for the entire set of proteins, select the top ranking $\left(\text{DCONT}\right)\times {\bar{N}}_{\text{cont}}$ edges, where ${\bar{N}}_{\text{cont}}$ is the average number of contact edges within the training set	0.5, 1, 1.5

The performance of the four graphs is similar, with a range of 0.002 in terms of PCC, 0.053 in terms of RMSE, 0.057 in terms of MAE, and 0.004 in terms of R^2^. Within these four metrics, the co-directionality graph performed best in terms of PCC, while the coordination graph performed best in terms of RMSE, MAE, and R^2^. For the co-directionality graph, this corresponds to a 0.02 increase in PCC compared to the contact graph. For the coordination graph, this corresponds to a 0.004 decrease in RMSE, a 0.038 decrease in MAE, and a 0.004 decrease in R^2^. On the other hand, the deformation graph had the worst performance across all metrics. This may be due to the high centrality of the deformation graph, as described in the previous section and shown in Figure 2. Furthermore, the contact graph had the same performance as the deformation graph in terms of PCC and R^2^. Overall, the three dynamical graphs all achieved similar performance to the contact graph, proving them to be viable alternatives to the contact graph as representations for proteins.

Multigraph representations combining all edge types are tested using all three feature extractors to screen for the best-performing combination. This was compared to three non-dynamical models: (1) contact with 12 Å cutoff paired with the S1 extractor; (2) backbone and contact graph paired with the M2 extractor; and (3) backbone and contact graph paired with the M1 extractor. The best model we obtained was based on a combination of contact, co-directionality, coordination, and deformation graph with the M2 feature extractor. The threshold was defined as the 1CONT, or the same number of edges for each entry as the contact graph with a 12 Å cutoff. It scored a PCC of 0.920, RMSE of 3.867, MAE of 2.992, and R^2^ of 0.845. In comparison to the first baseline model, there is a 0.003 increase in PCC, 0.069 decreases in RMSE, 0.042 decreases in MAE, and 0.006 increases in R^2^. It also outperforms the closest performing multigraph-based baseline model by 0.02 in PCC, 0.057 in RMSE, 0.036 in MAE, and 0.005 in R^2^. Overall, the multigraph representation with contact, co-directionality, coordination, and deformation graphs outperformed non-dynamical representations by a slight margin.

**Figure 3:**
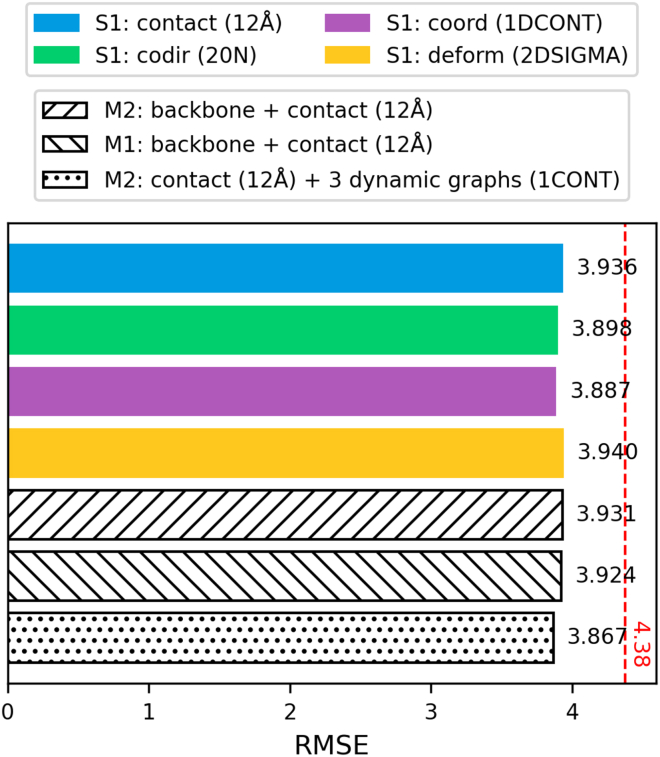
Comparison between the simple graph-based predictor trained on the 12 Å contact graph versus the best-performing co-directionality, coordination, and deformation graphs reveals that dynamical graphs are viable alternatives to contact graphs in the task of melting temperature prediction Performance of DeepSTABp on our test set is marked by the dashed line.

To assess whether the observed improvement of the best-performing multigraph model reflects a genuine performance gain rather than random fluctuations, we conducted additional statistical significance analyses to compare the best-performing multigraph model against the contact-only baseline. Specifically, bootstrap analysis with 10,000 resamples showed a mean difference of −0.0786, with a confidence interval narrowly overlapping zero (−0.1376 to 0.0002). Notably, the best-performing model outperformed the contact-only baseline model in 97.5% of evaluations. While the improvement is modest, this indicates that the observed gains are consistent across samples and unlikely to be due to random performance fluctuations. This supports the conclusion that incorporating dynamical graph information provides an incremental improvement over contact-only representations. A summary of model performance in terms of RMSE is given in Figure 3.

**Figure 4:**
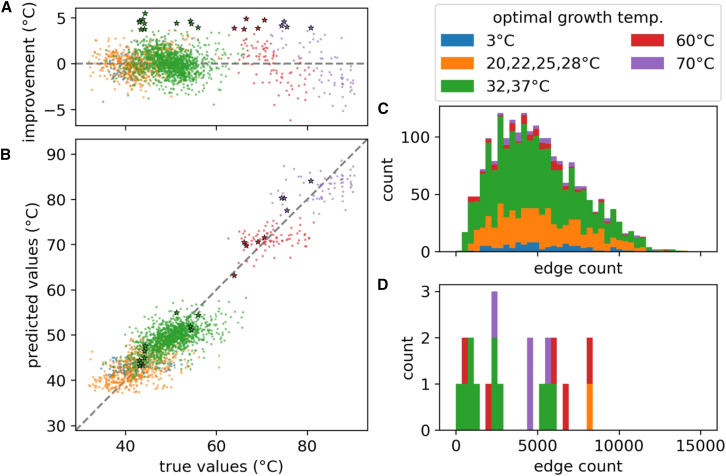
Analysis of the best-performing model based on dynamics-informed multigraph representations (A) The difference in absolute error between the contact graph baseline model and the best-performing model, grouped by OGT. (B) True vs. predicted values of the best-performing model show that the bias of the model is related to the OGT, where, within the same OGT bracket, it slightly underestimates the melting temperature for proteins with larger true values, and vice versa. In both figures, the top 20 proteins showing the most improvement are marked in red. (C) The edge count distribution of the test set, grouped by OGT. (D) The edge count distribution of the top 20 proteins. Compared to the entire set in (C), the edge counts are closer to 0.

The difference between the absolute error of the contact graph baseline model and of the best-performing model is plotted in Figure 4A. It is shown that the greatest improvement is around 5°C. The top 20 proteins showing the greatest improvements are also marked on the plot, where a large cluster is found around 45°C. Within the same optimal growth temperature (OGT) bracket, proteins with smaller melting temperatures tend to have larger improvements. Plotting the predicted values from the best-performing model against the true values in Figure 4B, we also found that the OGT has a visible influence on the predicted values. Specifically, within the same OGT bracket, the melting temperature for proteins with higher true values tends to be underestimated, and those with lower true values tend to be overestimated. The edge count distribution of the top 20 proteins is shown in Figure 4D. Compared to the distribution of the entire test set shown in Figure 4C, the improved proteins are geared closer to 0.

Together, these observations suggest that OGT functions as a strong global contextual feature that introduces a systematic bias in the predictions. Rather than overriding structural or dynamical features, OGT acts as a coarse-grained prior reflecting the thermal environment in which a protein evolves, anchoring predictions at the organism level. Within a given OGT bracket, finer-grained graph-based representations then modulate the final estimate, capturing intrinsic structural and dynamical determinants of thermostability. This interaction highlights an important modeling consideration: OGT encodes evolutionary and environmental constraints, whereas graph-derived features encode protein-specific properties. Disentangling and adaptively weighting these global and local contributions may represent a promising direction for future model refinement.

### Laplacian node centrality of coordination graphs aids the identification of potentially critical residues

**Figure 5:**
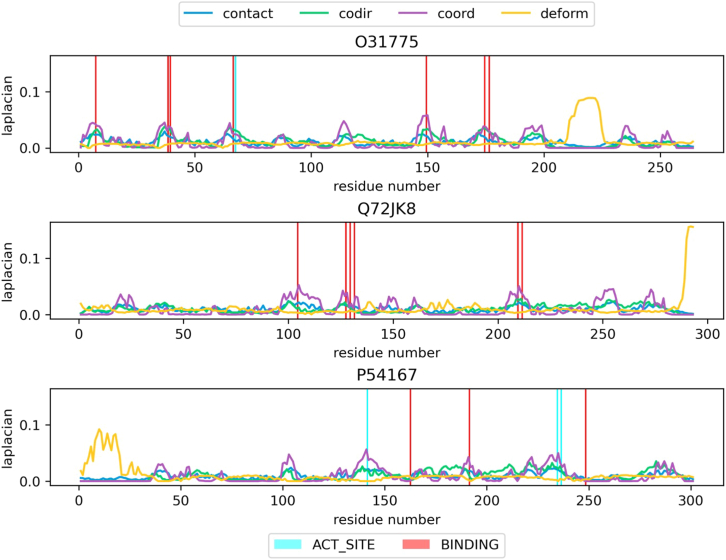
The Laplacian centrality of the coordination graph is an indicator of potentially critical residues To see this, the residue-level annotations from UniProt were plotted with the centrality along the sequence for UniProt: O31775 (phosphodiesterase), UniProt: Q72JK8 (ureohydrolase), and UniProt: P54167 (homoserine O-acetyltransferase). All residues annotated with ACT_SITE and BINDING in these proteins are marked, and most of the locations correspond well to the peak of coordination graphs. Of the four graphs, coordination has the clearest signal of potentially critical locations.

We took our best-performing setup and investigated how information regarding critical sites can be inferred from the representation. Juxtaposing node centrality along the sequence with residue-level annotations from UniProt revealed that the location of critical residues can be partially inferred from the Laplacian centrality of coordination graphs. This is shown in Figure 5 for dataset entries UniProt: O31775, UniProt: Q72JK8, and UniProt: P54167. O31775 is a phosphodiesterase of Bacillus subtilis. Residue 68 is annotated as an active site (ACT_SITE), defined as amino acids directly involved in the activity of an enzyme, and is noted as a “proton donor”, while residues 8, 39, 40, 67, 150, 175, and 177 are annotated as binding to the ferric cation. Q72JK8 is a ureohydrolase of Thermus thermophilus, and has residues 105, 128, 130, 132, 210, and 212 marked as binding to Mn^2+^. P54167 is a homoserine O-acetyltransferase of Bacillus subtilis. It is annotated as an active site at residue 142 with the note “Acyl-thioester intermediate”, at 235 with the note “proton acceptor”, and at 237 without any additional notes. Its residues 163, 192, and 249 are annotated as binding sites.

All the sites mentioned previously are located at or in the region of peaks of the Laplacian centrality for the coordination graph, except for residues 163 and 249 of P54167. Comparison between the four edge types shows that, despite having peaks and troughs at similar locations, the intensity of peaks from contact and co-directionality graphs is not as distinct from smaller peaks when compared to centrality computed based on coordination graphs. Therefore, we conclude that the Laplacian centrality of the coordination graph is the best indicator of potentially critical residues within the four edge types. This resonates with our finding in the previous section that the coordination graph achieved the best-performing melting temperature predictor of all four edge types.

Since high-centrality residues correspond to nodes with highly connected dynamical edges by definition, the residues highlighted based on coordination graphs likely exhibit coordinated motion with many other residues. It is also noteworthy that, in terms of Laplacian node centrality, the co-directionality and coordination graphs are more similar to the contact graph. Deformation graphs, on the other hand, exhibited a different distribution from the other three, often with one single main peak. This corresponds to the high centrality that was also observed during graph visualization, as seen in Figure 2.

Finally, we note that there are multiple cases where peaks in Laplacian centrality do not coincide with annotated sites. It is difficult to determine whether such discrepancies arise from incomplete functional annotations, which are known to be biased toward well-characterized proteins, or from limitations of the proposed graph representations. In certain cases, neighboring residues exhibit closely spaced peaks of varying magnitude, which can complicate interpretation. These observations indicate that the centrality analysis reveals enrichment tendencies and mechanistic signals rather than providing definitive functional site identification.

## Discussion

Our dynamics-informed approach to protein melting temperature prediction by introducing co-directionality, coordination, and deformation graphs as new representations of residue coupling provides a generic representation of protein dynamics. The results showed that these dynamical graphs alone achieved performance comparable to contact graphs, validating them as viable substitutes for conventional graph representations. Incorporating dynamic and static graphs into multigraph neural networks yielded measurable improvements over contact-only baselines, as confirmed by bootstrap analysis. We believe this combination of static and dynamic, as well as multigraph neural networks, can be generalized to other predictive and generative methods in protein design. Although the absolute RMSE gain of 0.07 seems modest, it represents a consistent improvement over baseline models across a large test set. Importantly, this boost is accompanied by interpretability and the demonstration that dynamical graphs can serve as viable alternatives to contact graphs—features that extend the significance of this work.

From a biological perspective, the interpretability analysis using Laplacian centrality on coordination graphs offers insights into how dynamic coupling relates to functional and stability-related residues. However, while residues with high centrality are near annotated catalytic or binding sites in some cases, this correspondence is not universal. Quantitative analysis indicates moderate recall and low precision, and allosteric sites are only partially captured. This reflects the limitation that dynamics-informed centrality shows network connectivity and collective mechanical coupling rather than definitive functional importance. The centrality analysis should therefore be interpreted as revealing enrichment tendencies and mechanistic signals, not as a direct predictor of functional residues.

The primary computational overhead of the proposed pipeline is from torsional network model (TNM)-based normal mode analysis, which is performed once per protein structure. Benchmarking across proteins ranging from approximately 250 to 1000 amino acids shows that TNM preprocessing scales superlinearly with protein length (1.8 s for 250 amino acids to 48.5 s for 1,000 amino acids), whereas graph construction incurs a comparatively modest cost (0.12 s for 250 amino acids to 1.08 s for 1,000 amino acids). In contrast, GNN inference is lightweight and nearly constant (0.12 s), indicating that prediction is not a runtime bottleneck. Peak memory usage remains within 400 MB even for larger proteins, suggesting that the method is feasible on standard workstation hardware.

Several directions emerge from this work. Extending dynamics-informed multigraph representations to other protein properties, such as activity, binding affinity, or mutational effects, could help clarify when and how different dynamical couplings are most informative. Methods for disentangling global contextual features, i.e., OGT, from intrinsic protein-specific dynamics may also enhance model interpretability and generalization. Combining dynamical graphs with generative models could be greatly beneficial for engineering protein stability and dynamical profiles.

In summary, our findings demonstrate that dynamics-informed multigraphs enrich protein representations and improve learning outcomes. While the performance gains are incremental, they are statistically meaningful, biologically interpretable, and achieved with acceptable computational cost. More broadly, this framework provides a generalizable strategy for incorporating dynamics into protein ML pipelines, with implications for thermostability prediction and diverse protein properties.

### Limitations of the study

Some methodological limitations should be acknowledged. First, the deformation graph consistently underperforms other dynamical edge types. Its highly centralized connectivity suggests that deformation edges emphasize global mechanical responses dominated by a small subset of residues, which may lead to it being less informative on global behavior. In this sense, it might be more suitable for tasks such as the identification of mechanically sensitive regions. Second, threshold selection for graph construction, while systematic, remains empirical. Although our two-stage screening procedure demonstrates robustness across a large search space, future work could explore adaptive or learned thresholding schemes. Finally, limitations arise from the exclusion of proteins lacking AlphaFold structures or for which TNM normal mode analysis could not be completed. Our analysis shows that these missing entries are predominantly longer proteins, leading to all proteins being shorter than 1,091 amino acids in the final dataset. While melting temperature distributions remain largely unchanged, the truncation of sequence length distribution introduces a length-related bias that may limit model generalization to larger proteins. This bias is primarily driven by current constraints in AlphaFoldDB coverage and the computational scalability of TNM rather than by the proposed modeling framework itself. Optimization of current solvers or incorporation of more scalable elastic network models (ENMs) would accommodate larger proteins. The melting temperature and sequence length distribution of four sets: (1) the original DeepSTABp-lysate dataset, (2) the final curated dataset, (3) proteins lacking AlphaFold structures, and (4) proteins for which TNM failed, are given in Figure S11.

## Resource availability

### Lead contact

Requests for further information and resources should be directed to and will be fulfilled by the lead contact, Dr. Shu-Wei Chang (changsw@ntu.edu.tw).

### Materials availability

This study did not generate new unique reagents.

### Data and code availability

• All data reported in this paper will be shared by the lead contact upon request.
• All original code for downloading AlphaFold structures, computation and generation of the graph representations, training and evaluation of the GNN models, analysis, and visualization has been deposited at ZENODO and is publicly available as of the date of publication. The DOI is listed in the key resources table.
• Any additional information required to reanalyze the data reported in this paper is available from the lead contact upon request.

## Acknowledgments

This study was funded by 10.13039/501100006477National Taiwan University (NTU-114L7846) and the 10.13039/100020595National Science and Technology Council (10.13039/100020595NSTC 112-2628-E-002-014-MY3 and 114-2223-E-002-006-MY3). We also thank the National Center for Research on Earthquake Engineering for providing computational resources.

## Author contributions

Conceptualization, methodology, software, validation, investigation, data curation, writing – original draft, writing – review and editing, visualization, Y.-L.C.; conceptualization, resources, writing – review and editing, supervision, project administration, Funding Acquisition, S.-W.C.

## Declaration of interests

The authors declare no competing interests.

## Declaration of generative AI and AI-assisted technologies in the writing process

During the preparation of this work, the author(s) used ChatGPT to assist with language polishing. After using this tool or service, the author(s) reviewed and edited the content as needed and take(s) full responsibility for the content of the publication.

## STAR★Methods

### Key resources table

**Table:** 

REAGENT or RESOURCE	SOURCE	IDENTIFIER
**Software and algorithms**		

This work	Version 1.0.0	https://doi.org/10.5281/zenodo.19548763
ProDy	Version 2.6.1	https://github.com/prody/ProDy/releases
PyTorch	Version 2.8.0	https://github.com/pytorch/pytorch/releases
PyG	Version 2.6.1	https://github.com/pyg-team/pytorch_geometric/releases
AlphaFold Protein Structure Database	Version 6	https://alphafold.ebi.ac.uk

### Method details

#### Dynamics-informed graph representations

Elastic network models (ENMs) are coarse-grained mechanical frameworks frequently used in normal mode analysis to probe protein dynamics from static structures. In this work, we used the TNM^35^ as the surrogate mechanical model. Co-directionality, coordination, and deformation couplings introduced by Alfayate et al.^36^ were computed based on the normal modes to quantify protein dynamics. Since the coupling values are defined for each pair of residues in the protein, the aim was to summarize these couplings succinctly into graphs by applying thresholds, analogous to our previous work.^21^ An overview of the data processing procedure is given in Figure S30.

In essence, co-directionality coupling captures the propensity of residues to move in the same direction. With the frequency denoted by $\omega_{\alpha }^{2}$ and the mode shape of the *α*-th normal mode denoted by ${\vec{x}}^{\alpha }$, co-directionality coupling is computed as

(Equation 1) $$\begin{array}{c} C_{ij}^{codir}=\frac{\sum_{\alpha }\frac{1}{\omega_{\alpha }^{2}}\frac{{\vec{x}}_{i}^{\alpha }}{\left|{\vec{x}}_{i}^{\alpha }\right|}\frac{{\vec{x}}_{j}^{\alpha }}{\left|{\vec{x}}_{j}^{\alpha }\right|}}{\sum_{\alpha }\frac{1}{\omega_{\alpha }^{2}}} \end{array}$$

with ${\vec{x}}_{i}^{\alpha }$ being the 3-dimensional vector of motion for the *i*-th residue in the mode shape. Note that the weighted average with $1/\omega_{\alpha }^{2}$ is equivalent to the equipartition theorem, as each degree of freedom should take the same amount of energy. Coordination coupling captures the propensity of residue pairs to move in unison, i.e., with constant distance, and is computed as

(Equation 2) $$\begin{array}{c} C_{ij}^{coord}=1-0.5\sqrt{\sum_{\alpha }\frac{1}{\omega_{\alpha }^{2}}{\left(\frac{{\vec{r}}_{i}^{0}-{\vec{r}}_{j}^{0}}{\left|{\vec{r}}_{i}^{0}-{\vec{r}}_{j}^{0}\right|}\cdot \left({\vec{x}}_{i}^{\alpha }-{\vec{x}}_{j}^{\alpha }\right)\right)}^{2}} \end{array}$$

where ${\vec{r}}_{j}^{0}$ is the position of the *j*-th residue in equilibrium. The parameters 1 and 0.5 are designed to keep the output mostly positive, as stated in the original work.^36^ Deformation coupling is defined in a slightly different flavor from the two coupling types above. It quantifies the magnitude of deformation that can be induced on the *i*-th residue when a unit perturbation force $\vec{f}$ is applied to the *j*-th residue. It is computed as

(Equation 3) $$C_{ij}^{deform}=\max\left\{{\left|{\vec{r}}_{i}-{\vec{r}}_{i}^{0}\right|}^{2}\;|\;\vec{f}\;\text{applied}\;\text{to}\;\text{the}\;j-\text{th}\;\text{residue}\right\}\begin{array}{c} =\max\left\{\vec{f}\cdot {\left(F^{\left(ij\right)}\right)}^{T}F^{\left(ij\right)}\cdot \vec{f}\right\} \end{array}=\max\left\{\text{eigenval}\left({\left(F^{\left(ij\right)}\right)}^{T}F^{\left(ij\right)}\right)\right\}$$

where $F_{mn}^{\left(ij\right)}=\sum_{\alpha }x_{im}^{\alpha }x_{in}^{\alpha }/\omega_{\alpha }^{2}$, with $\left(x_{ix}^{\alpha },x_{iy}^{\alpha },x_{iz}^{\alpha }\right)$ being the components of the eigenvector ${\vec{x}}^{\alpha }$ in Cartesian coordinates. Note that the couplings are all designed to be symmetric, i.e., $C_{ij}=C_{ji}$, by definition. We used the official implementation on GitHub (github.com/ugobas/tnm) to compute these couplings.

A threshold must be chosen to build a graph from the pairwise coupling values, similar to the cutoff for contact graphs. This can be a universal value applied across all proteins in the dataset or one that is computed and tailored to individual proteins. Since values too small result in a fully connected graph and values too large result in an edgeless graph, we expected the existence of an adequate threshold would give the most informative representation. We tested various threshold definitions for each graph as laid out in the next section.

ProtTrans embeddings^15^ were used as the node features. Specifically, we fed the protein sequence into the pre-trained NLP model, where an n-residue sequence generates an (n+2)×1024 embedding. The 2 additional vectors correspond to the start and end tokens, which were discarded. The remaining n vectors of 1024 dimensions were used as the node features. Lastly, the OGT was also used as an input to our model due to its high correlation with melting temperature.

#### Threshold definitions

A threshold value is required to convert the symmetric, fully-connected coupling matrix into a simple graph. We outline the seven definitions for the threshold value that were tested in Table 1. For N, SIGMA, CONT, and PAIR, the threshold values were determined for each protein independently from other proteins. The training dataset was considered when computing the threshold value for DN, DSIGMA, and DCONT. All proteins in both the training and test sets shared the same threshold value in the latter three. Note that the threshold definition can be designed to differ from edge type to edge type, or even from protein to protein. For simplicity and to save computational resources, we limited our search to cases where the same definition is shared across all proteins in the dataset. We also only considered cases where each dynamical edge type shares the same definition when building our multigraph representations. All graph representations built from these definitions are reported in Table S1.

#### Dataset and Data Processing

We adopted the dataset used in DeepSTABp^14^ for this work. Specifically, we took the “lysate” branch of their dataset with 25,938 proteins for data processing and the corresponding melting temperature as labels for training and testing. Starting with the UniProt accessions given in their source code, we downloaded protein structures from AlphaFoldDB^37^ for TNM analysis. There were two points of failure in this process: the structure for some accessions might not be available in AlphaFoldDB, and singularities in normal mode analysis with TNM. We discarded any proteins that were not processed successfully. This left us with 25,445 proteins with structures that were fed into the TNM program, where another 3,463 proteins were discarded due to errors in processing. We also adopted their data split, with the exception of the discarded proteins. The resulting training set had 19,938 entries, and the test set had 2,044 entries.

Additionally, a random selection of 896 entries from the training dataset was used to speed up the representation selection. This preliminary set was used to screen for the best setup while reducing the required computational power. It also served as a surrogate of the full training set for computing dataset-wide thresholds. Information on the training procedure is given in the section on Training Procedure below.

#### GNN architecture

Three architectures for graph feature extractors were used in this work: S1, M1, and M2. The S1 architecture consisted of a single GCN layer and a max-pooling layer to extract information from simple graphs, with the output node feature size set to 32. The M1 and M2 architecture, on the other hand, was designed to take advantage of multigraph features, where various sets of edge connections share the same set of nodes. M1 consisted of multiple independent GCN blocks, each comprised of one GCN layer and meant to deal with one type of edge connections. The node features from each GCN block were then concatenated and max-pooled to form graph-level features. The feature size of each GCN block was set to 32. M2 made use of the multi-dimensional graph convolutional network (mGCN),^32^ a graph aggregation designed specifically to process multigraphs by allowing for crosstalk between the aggregation of individual edge types. The output from mGCN was also sent through a max-pooling layer to form the graph-level feature. The output feature size of the mGCN layer was set to 32 as well.

In addition to the graph, input to the model also included the OGT, as this value is known to correlate highly with the melting temperature. We used a simple neural network to extract the OGT feature. This feature extractor consisted of 2 neural layers of width 20 and 10, each followed by an activation layer. Finally, the OGT feature and the graph-level feature were concatenated and fed into the final neural network predictor to make the temperature prediction. This module consisted of 3 neural layers with a linear width reduction from the input width to 1. The initial 2 layers were followed by an activation layer and a batch normalization layer, and the final neural layer had no successors. All activation layers were leaky ReLUs with a slope of 0.01. The various components included in this work are summarized in Figure S31.

We briefly describe the mGCN aggregation here. Detail-oriented readers are referred to the original article^32^ for a formal derivation, where a visualization of the framework is given in Figure 3 of the publication. Adhering to the terminology of the original paper, the node features used as input to an mGCN layer are referred to as the general representation. Denoting the general representation in matrix form as H, it is multiplied by the projection matrix and passed through a non-linear activation function to produce the dimension-specific representation, i.e.,

(Equation 4) $$\begin{array}{c} E_{d}=\sigma \left(W_{d}\;\cdot \;H\right) \end{array}$$

where $W_{d}$ denotes the projection matrix, $E_{d}$ denotes the dimension-specific representation of the dimension d, and σ denotes the non-linear activation function. The dimension-specific representation is then sent through the within-dimension aggregation and the across-dimension aggregation separately. The within-dimension aggregation is computed as

(Equation 5) $$\begin{array}{c} {H_{w}}_{d}=E_{d}\;\cdot \;{\hat{A}}_{d} \end{array}$$

where ${\hat{A}}_{d}$ denotes the row-normalized adjacency matrix for dimension d, and ${H_{w}}_{d}$ denotes the within-dimension aggregated node features of dimension d. The across-dimension aggregation is computed as the weighted sum of the various dimension-specific representations, or

(Equation 6) $$\begin{array}{c} {H_{a}}_{d}=\sum_{g=1,2,\ldots ,D}b_{g,d}\;\cdot \;E_{g} \end{array}$$

where $E_{g}$ denotes the dimension-specific representation of the dimension g, ${H_{a}}_{d}$ denotes the across-dimension aggregated node features, and $b_{g,d}$ denotes the weighting computed as

(Equation 7) $$\begin{array}{c} b_{g,d}=\text{Softmax}\left(p_{g,d}\right)=\frac{\exp\left(p_{g,d}\right)}{\sum_{g=1}^{D}\exp\left(p_{g,d}\right)} \end{array}$$

with

(Equation 8) $$\begin{array}{c} p_{g,d}=\text{tr}\left({W_{g}}^{T}M^{k}W_{d}\right) \end{array}$$

where $M^{k}$ denotes a trainable bilinear matrix to be learned, $W_{g}$ and $W_{d}$ denote the projection matrix for dimensions g and d, respectively, and tr(·) denotes the trace function. The new dimension-specific representation of dimension d is computed as the mean of the within-dimension aggregated node features ${H_{w}}_{d}$ and the across-dimension aggregated node features ${H_{a}}_{d}$

(Equation 9) $$\begin{array}{c} H_{d}=0.5\;\cdot \;\left({H_{w}}_{d}+{H_{a}}_{d}\right) \end{array}$$

where $H_{d}$ denotes the new dimension-specific representation. Finally, the new general representation is computed by concatenating the node features across dimensions and projecting them with matrix multiplication and a non-linear activation, or

(Equation 10) $$\begin{array}{c} H^{\prime }=\sigma \left(W\;\cdot \;{\text{Concat}}_{d=1}^{D}H_{d}\right) \end{array}$$

where W is a trainable matrix and $H^{\prime }$ denotes the new general representation. mGCN was implemented in-house with PyG^38^ in Python.

#### Training procedure

We adopted a two-stage screening procedure to reduce computational cost while systematically exploring the threshold configurations defined in Table 1. In Stage 1, models were trained using a preliminary dataset of 896 proteins (see dataset and data processing) to efficiently evaluate a large space of candidate graph representations. Specifically, we exhaustively constructed 63 simple dynamical graphs corresponding to all combinations of the seven threshold definitions listed in Table 1, three parameter values per definition, and three dynamical edge types. These dynamical edges were further combined with backbone edges, contact edges, or both, yielding 189 multigraph representations. In addition, multigraphs incorporating all three dynamical edge types simultaneously were evaluated with four configurations: backbone only, contact only, both, or neither, resulting in an additional 84 multigraph representations. In total, 336 graph representations (63 simple graphs and 273 multigraphs) were evaluated in Stage 1. A summary of these configurations is provided in Table S1.

Prior to model training, graph representations with insufficient connectivity (fewer than 10 edges on average across the preliminary dataset) were excluded based on edge statistics. For the remaining representations, one model with the S1 extractor was trained for each simple graph, while two models corresponding to the M1 and M2 extractors were trained for each multigraph representation, resulting in a total of 601 trained models in Stage 1. Models were trained using 10-fold cross-validation, and for each fold, the epoch with the best validation performance was selected as the representative model. The best-performing representative model across folds was then evaluated on the test set to produce the ranking.

The best-performing representations from Stage 1 were selected for the next stage. In this phase, graph representations were built for the full training dataset of 19,938 proteins and retrained using the same training and evaluation protocol. For dataset-wide thresholds (DN, DSIGMA, and DCONT), threshold values computed on the preliminary dataset were reused in Stage 2. The final results reported in the Results section correspond to this full-dataset evaluation. Performance statistics for all evaluated configurations are provided in Figures S12–S29.

Across both stages, melting temperature values were normalized independently such that training-fold values lay between 0 and 1. All models were trained with PyTorch^39^ using the AdamW optimizer with default weight decay (0.01) and betas (0.9, 0.999), an initial learning rate of 0.01, 30 training epochs, a batch size of 64, and no learning-rate schedulers.

#### Node centrality metrics

The Laplacian centrality^40^ of nodes was analyzed to investigate the feasibility of identifying critical residues based on the graph connections. The Laplacian centrality of a node is equal to the reduction in the Laplacian energy after excluding the node from the graph. Mathematically, the Laplacian centrality $C_{L}$ for node $v_{i}$ within graph G(V,E) is computed as

(Equation 11) $$\begin{array}{c} C_{L}\left(G,v_{i}\right)=\frac{U_{L}\left(G\right)-U_{L}\left(G_{i}\right)}{U_{L}\left(G\right)} \end{array}$$

where $G_{i}$ denotes the resulting graph after removal of $v_{i}$ from G, and $U_{L}\left(G\right)$ denotes the Laplacian energy of G, which is computed as

(Equation 12) $$\begin{array}{c} U_{L}\left(G\right)=\sum_{i=0}^{n}\lambda_{i}^{2} \end{array}$$

with n being the number of nodes in G and λ the eigenvalues of the Laplacian matrix of G.

This analysis was conducted using NetworkX.^41^

### Quantification and statistical analysis

All statistical analyses and model evaluations were conducted in Python using PyTorch^39^ and NumPy. While model evaluation was primarily based on direct comparisons of RMSE on the test set, the PCC between predicted and true melting temperature was also reported. Exact values of test performance in terms of PCC are provided in the main text, where the sample size (*n*) is uniformly the size of the test set, or 2,044.
