# Supplementary material for: Dynamics-informed multigraph neural networks for protein thermostability prediction and residue-level interpretation
Source: iScience. 2026 May 25;29(6):116089. doi: 10.1016/j.isci.2026.116089 (PMC13226212; doi:10.1016/j.isci.2026.116089)
Supplement: Document S1. Figures S1–S31 and Table S1 [file mmc1.pdf]

iScience, Volume *29*

Supplemental information

Dynamics-informed multigraph neural networks for protein thermostability prediction and residue-level interpretation

Yen-Lin Chen and Shu-Wei Chang

Table S1. Dynamical representations tested. A total of 63 simple graph representations (=5x7x3, rows 1 to 3) and 274 multigraph representations (=13x7x3+1, rows 4 to 17) were built and tested. Note that each row translates to 21 graph representations.

#	**Backbone**	**Contact**	**Codir**	**Coord**	**Deform**
1	—	—	all 7x3 definitions tested	—	—
2	—	—	—	all 7x3 definitions tested	—
3	—	—	—	—	all 7x3 definitions tested
4	—	—	all 7x3 definitions tested	definition same as codir	definition same as codir
5	v	—	all 7x3 definitions tested	—	—
6	v	—	—	all 7x3 definitions tested	—
7	v	—	—	—	all 7x3 definitions tested
8	v	—	all 7x3 definitions tested	definition same as codir	definition same as codir
9	v	12 Å	all 7x3 definitions tested	—	—
10	v	12 Å	—	all 7x3 definitions tested	—
11	v	12 Å	—	—	all 7x3 definitions tested
12	—	12 Å	all 7x3 definitions tested	—	—
13	—	12 Å	—	all 7x3 definitions tested	—
14	—	12 Å	—	—	all 7x3 definitions tested
15	—	12 Å	all 7x3 definitions tested	definition same as codir	definition same as codir
16	v	12 Å	all 7x3 definitions tested	definition same as codir	definition same as codir

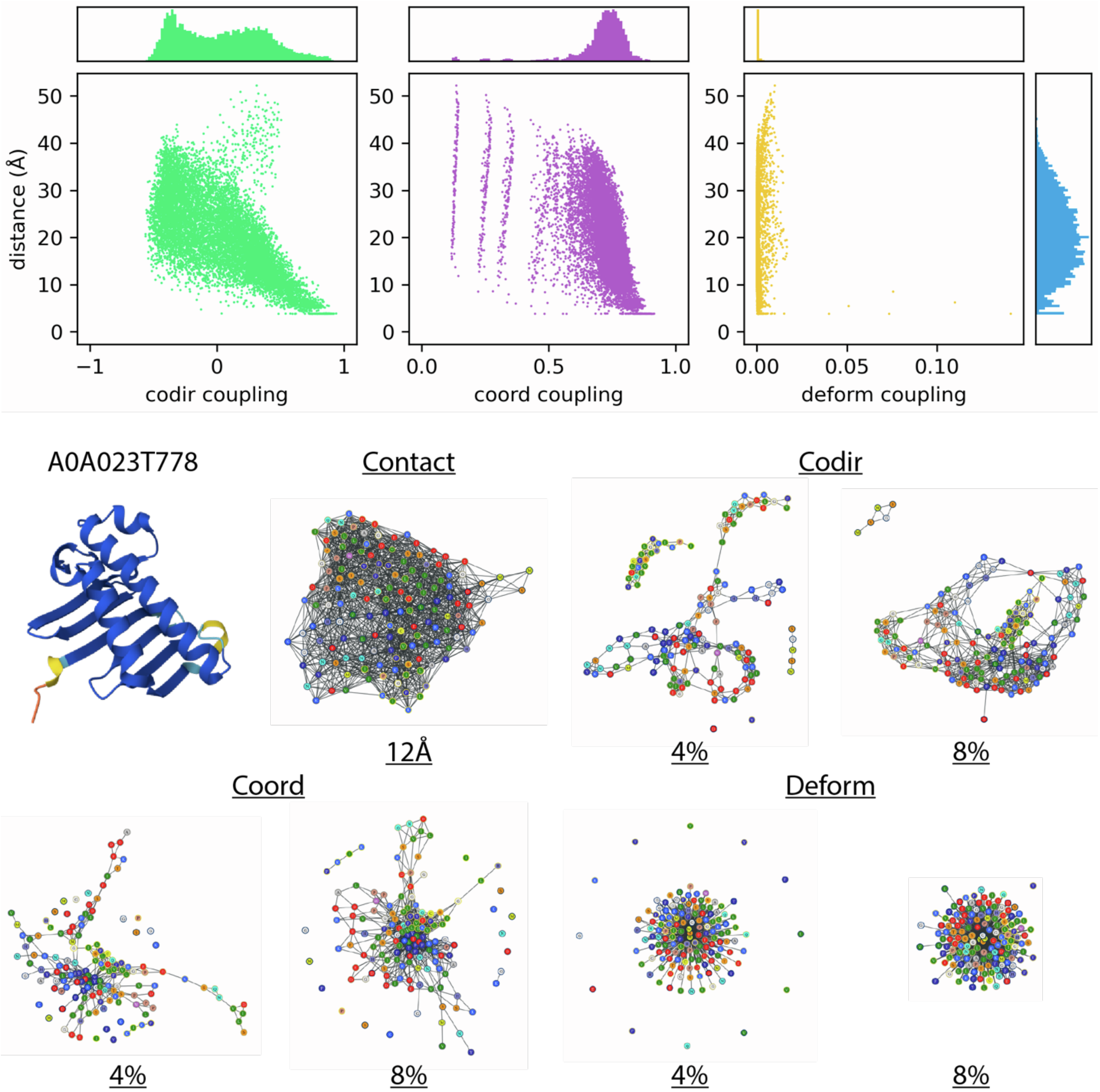

Figure S1. Correlation between coupling values and graph visualization using the scalable force-directed placement (SFDP) layout for UniProt accession A0A023T778.

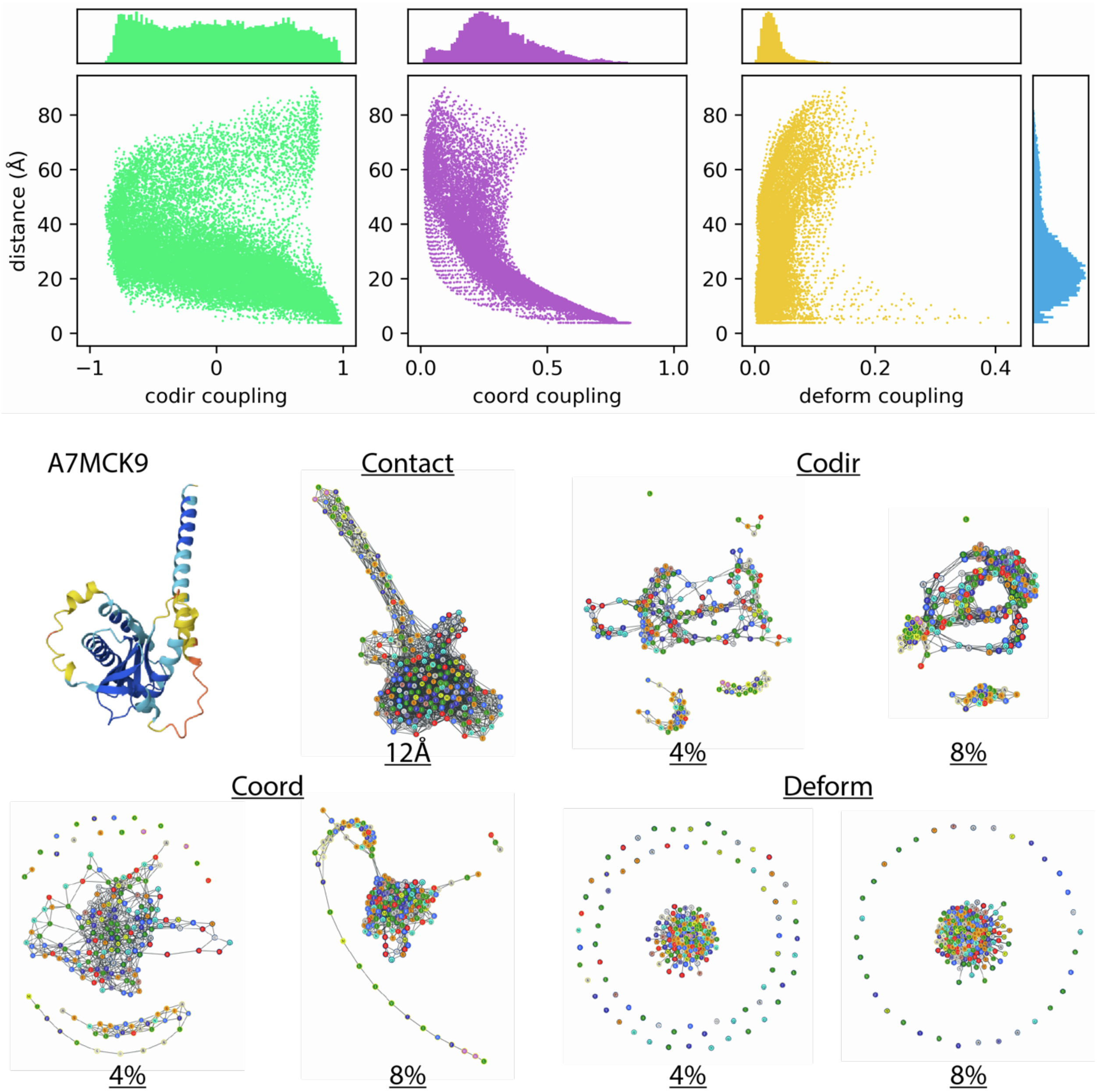

Figure S2. Correlation between coupling values and graph visualization using the scalable force-directed placement (SFDP) layout for UniProt accession A7MCK9.

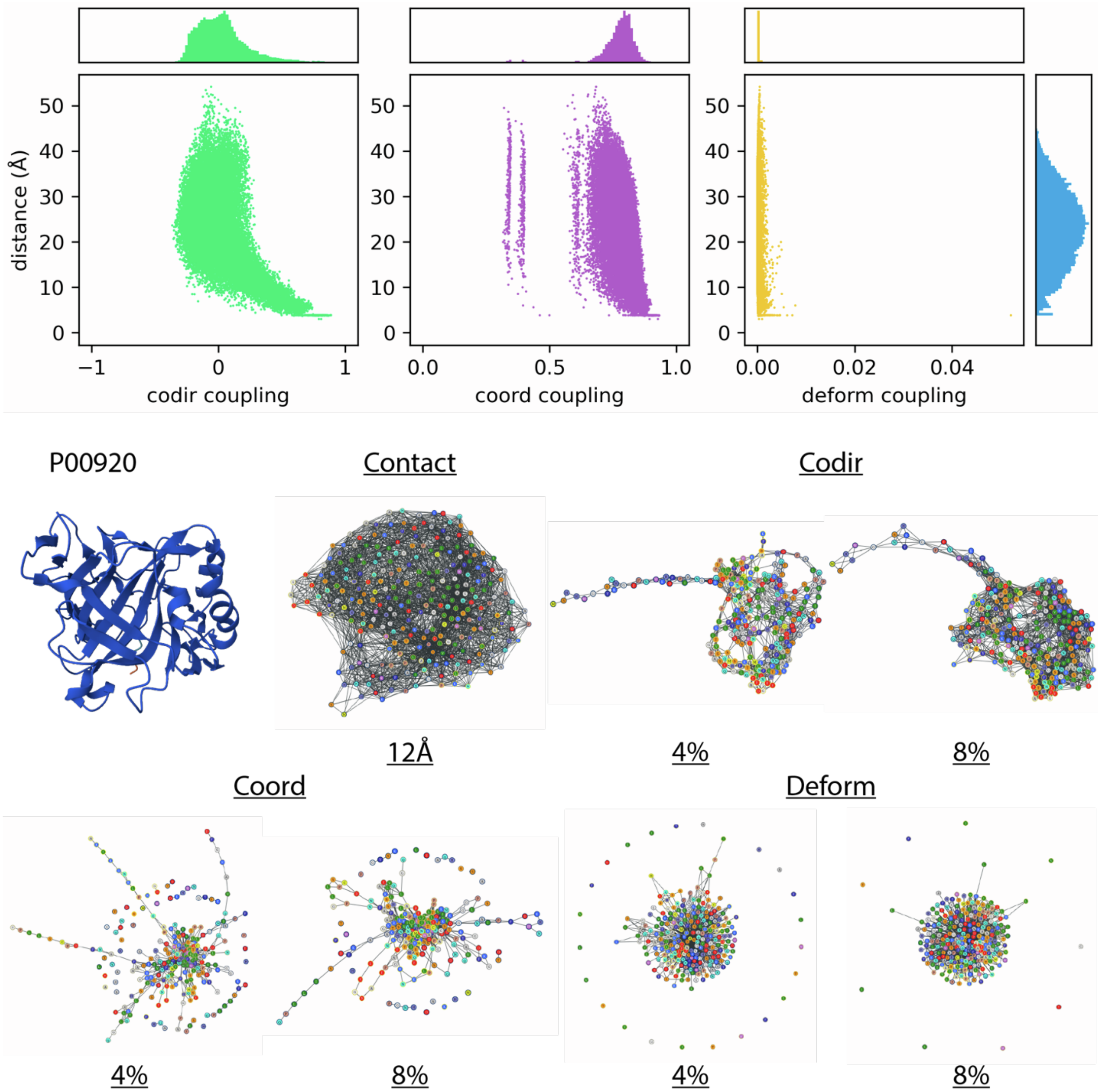

Figure S3. Correlation between coupling values and graph visualization using the scalable force-directed placement (SFDP) layout for UniProt accession P00920.

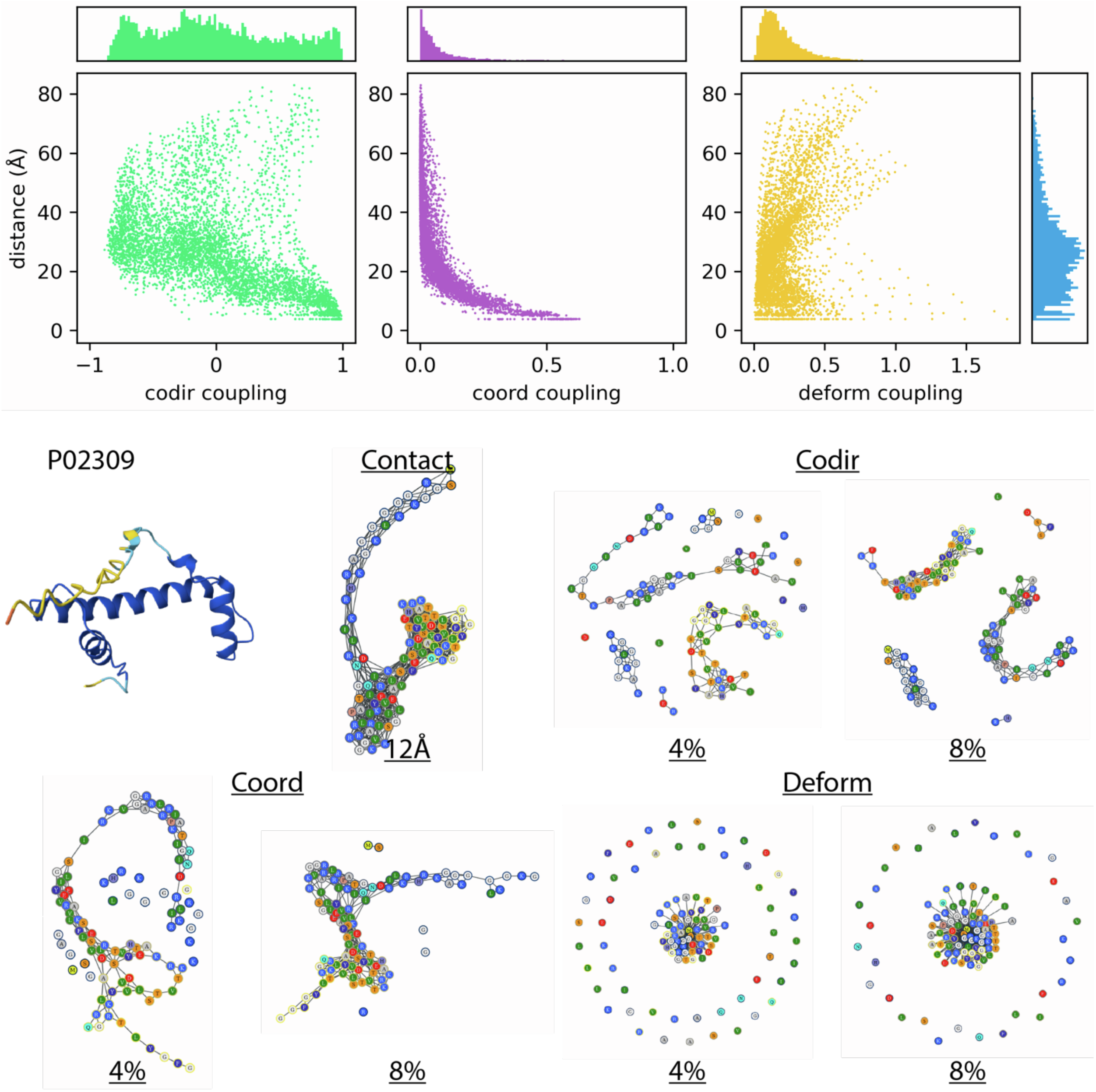

Figure S4. Correlation between coupling values and graph visualization using the scalable force-directed placement (SFDP) layout for UniProt accession P02309.

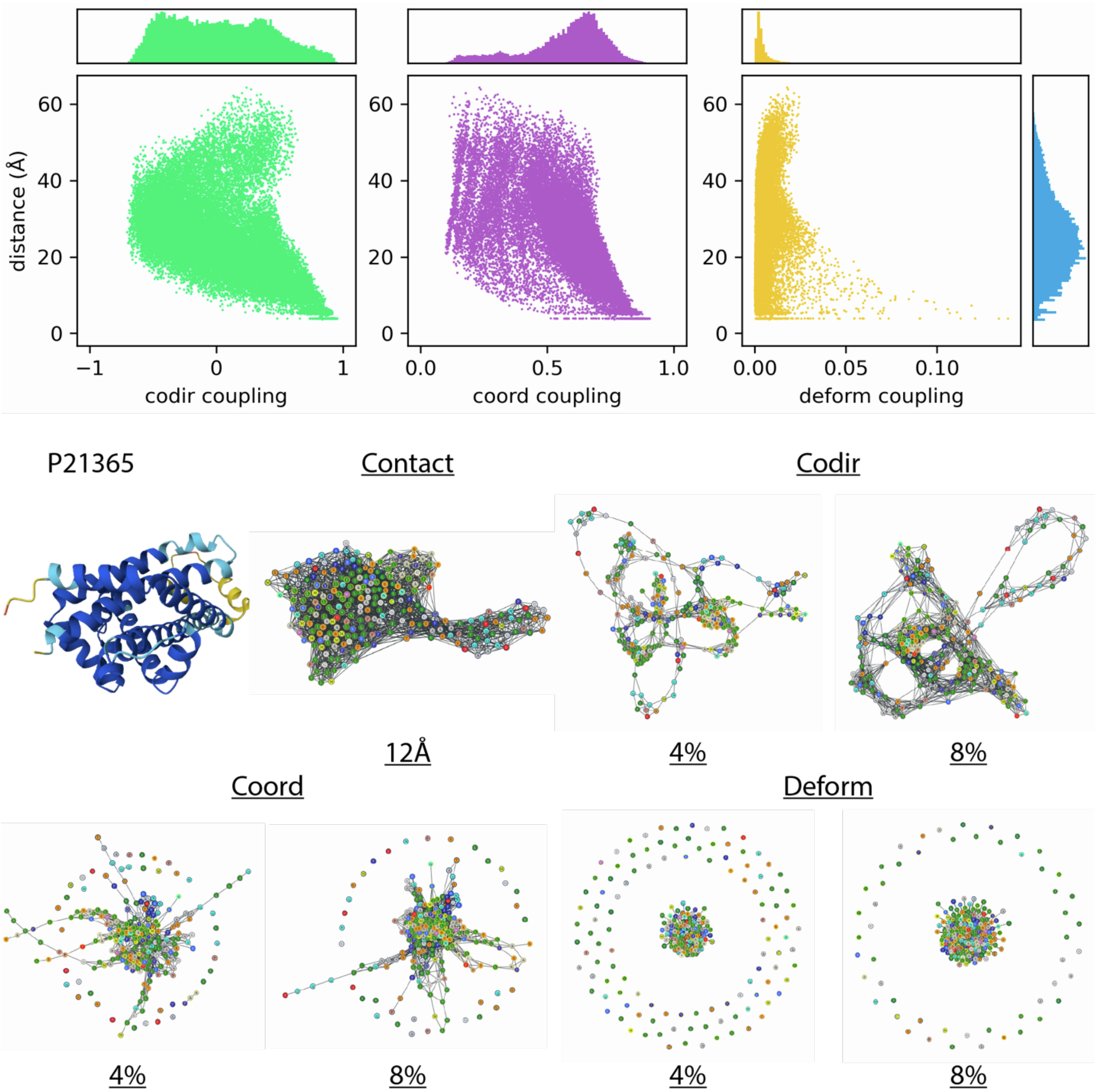

Figure S5. Correlation between coupling values and graph visualization using the scalable force-directed placement (SFDP) layout for UniProt accession P21365.

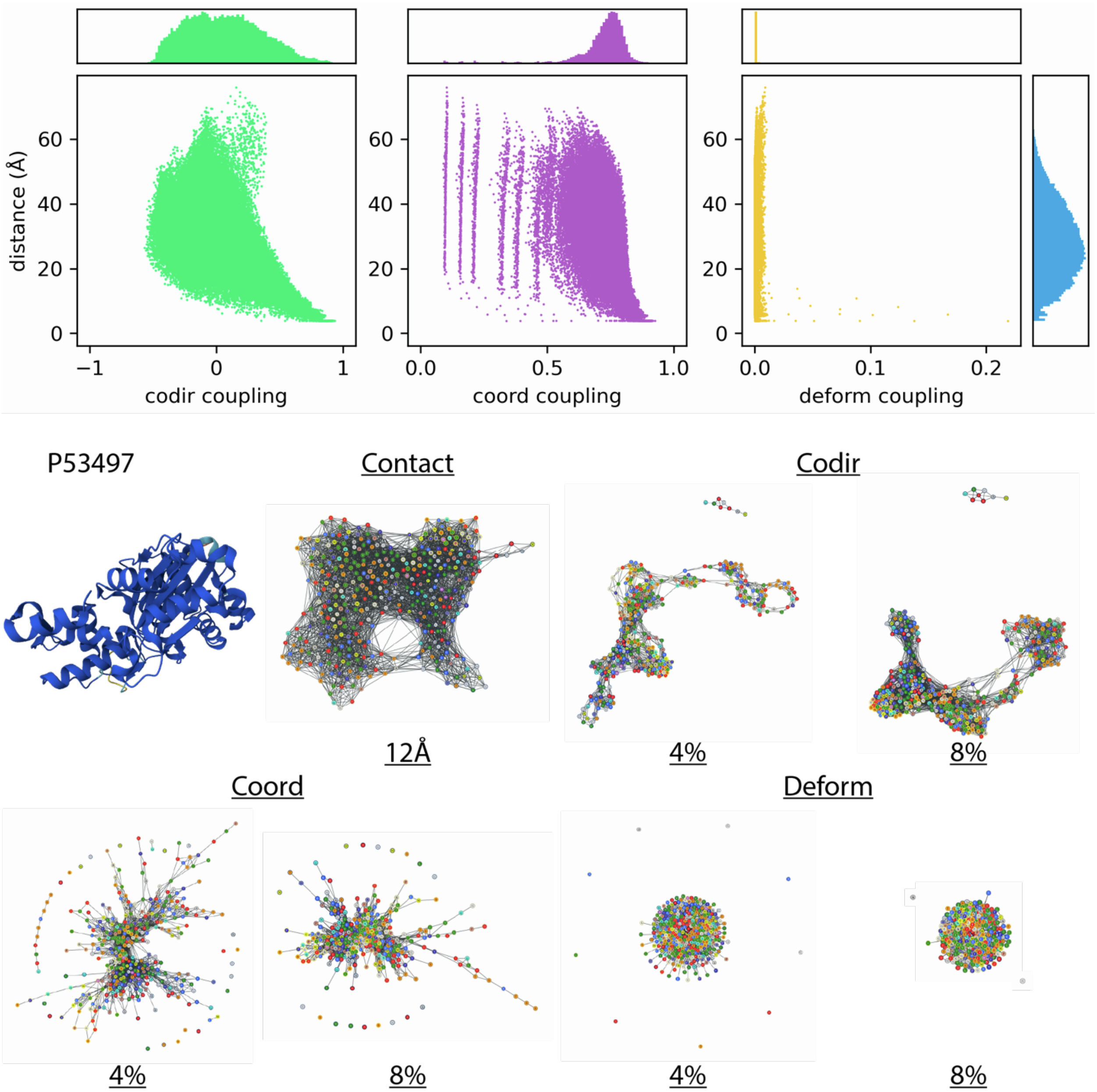

Figure S6. Correlation between coupling values and graph visualization using the scalable force-directed placement (SFDP) layout for UniProt accession P53497.

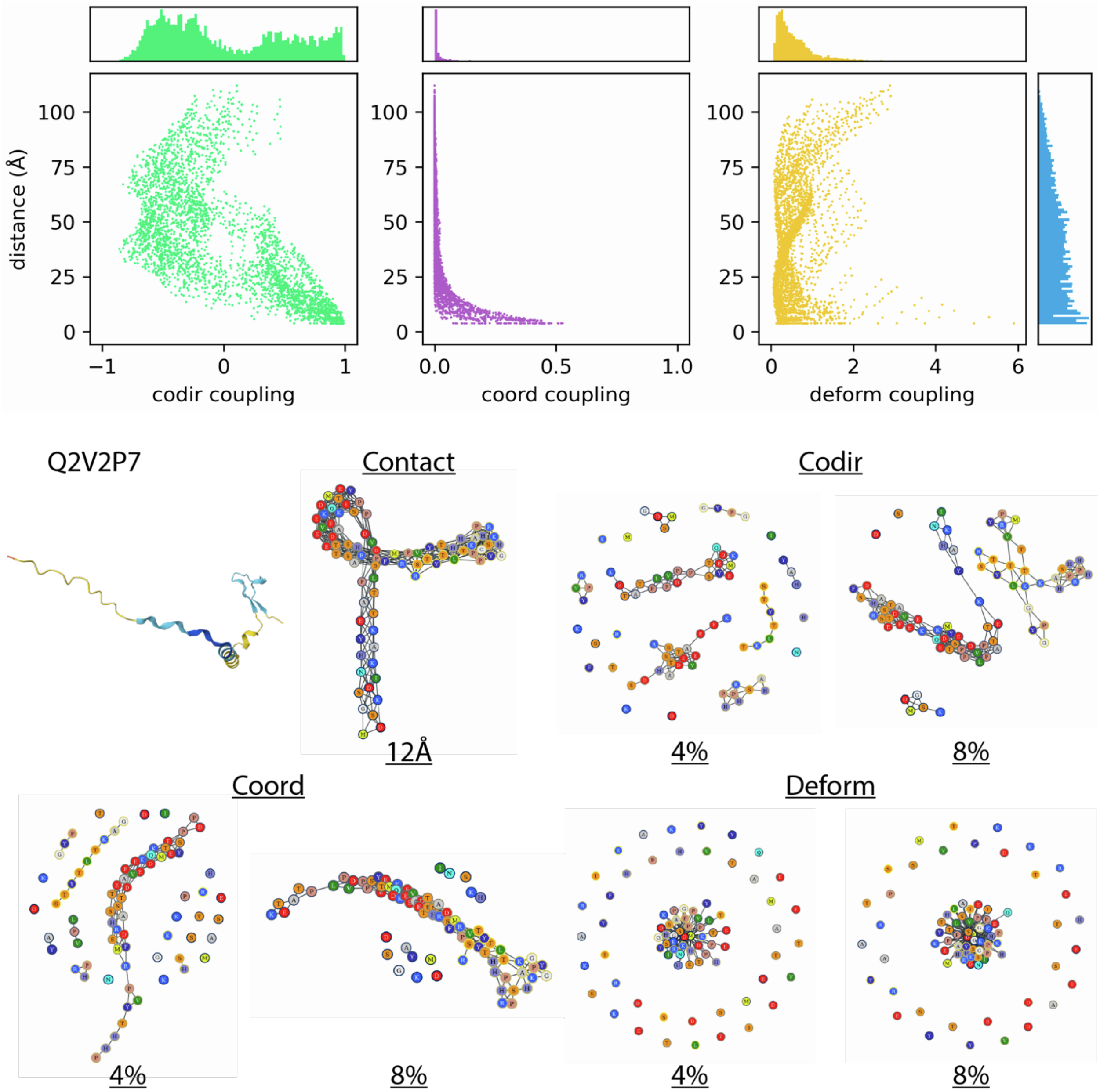

Figure S7. Correlation between coupling values and graph visualization using the scalable force-directed placement (SFDP) layout for UniProt accession Q2V2P7.

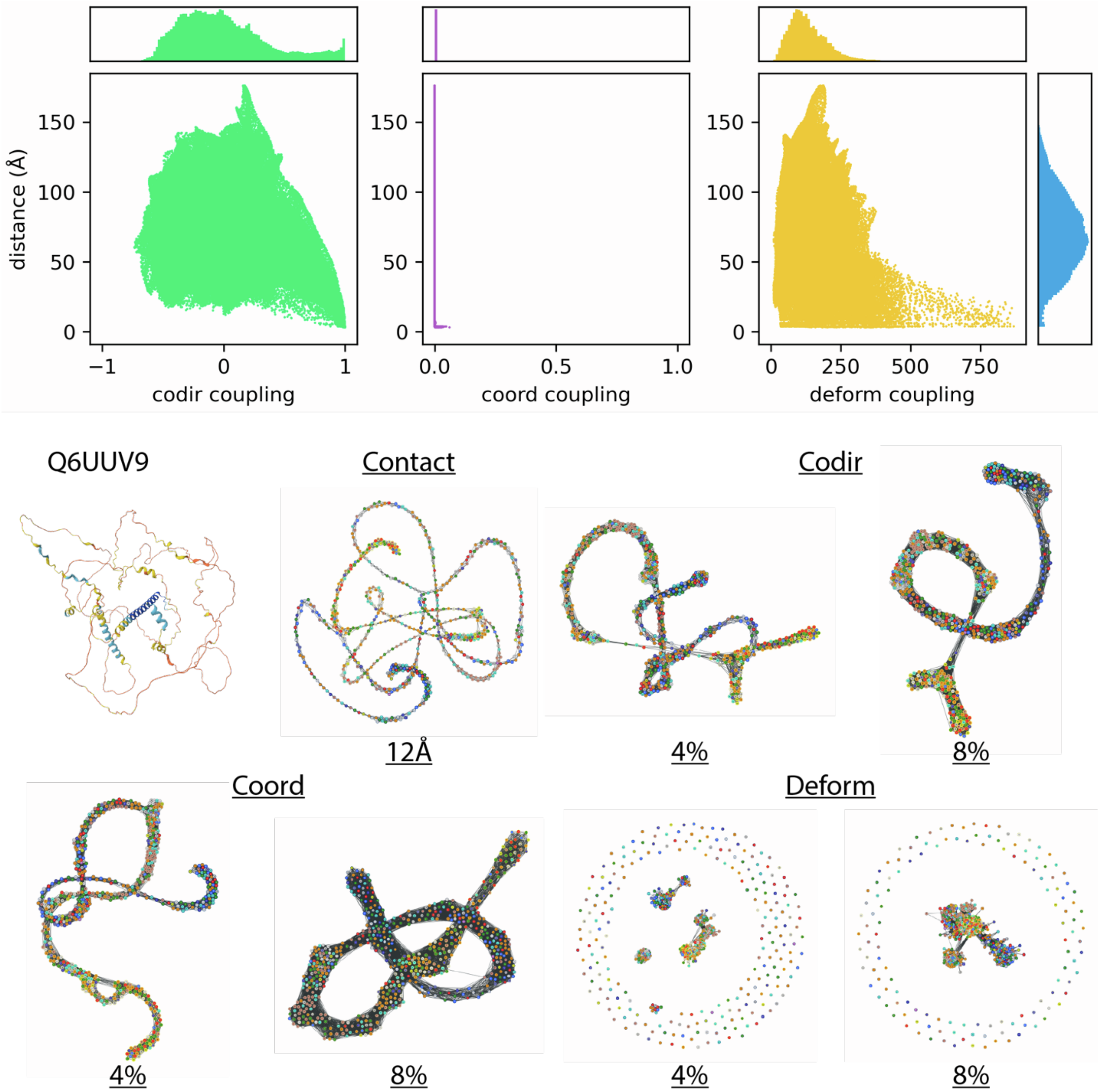

Figure S8. Correlation between coupling values and graph visualization using the scalable force-directed placement (SFDP) layout for UniProt accession Q6UUV9.

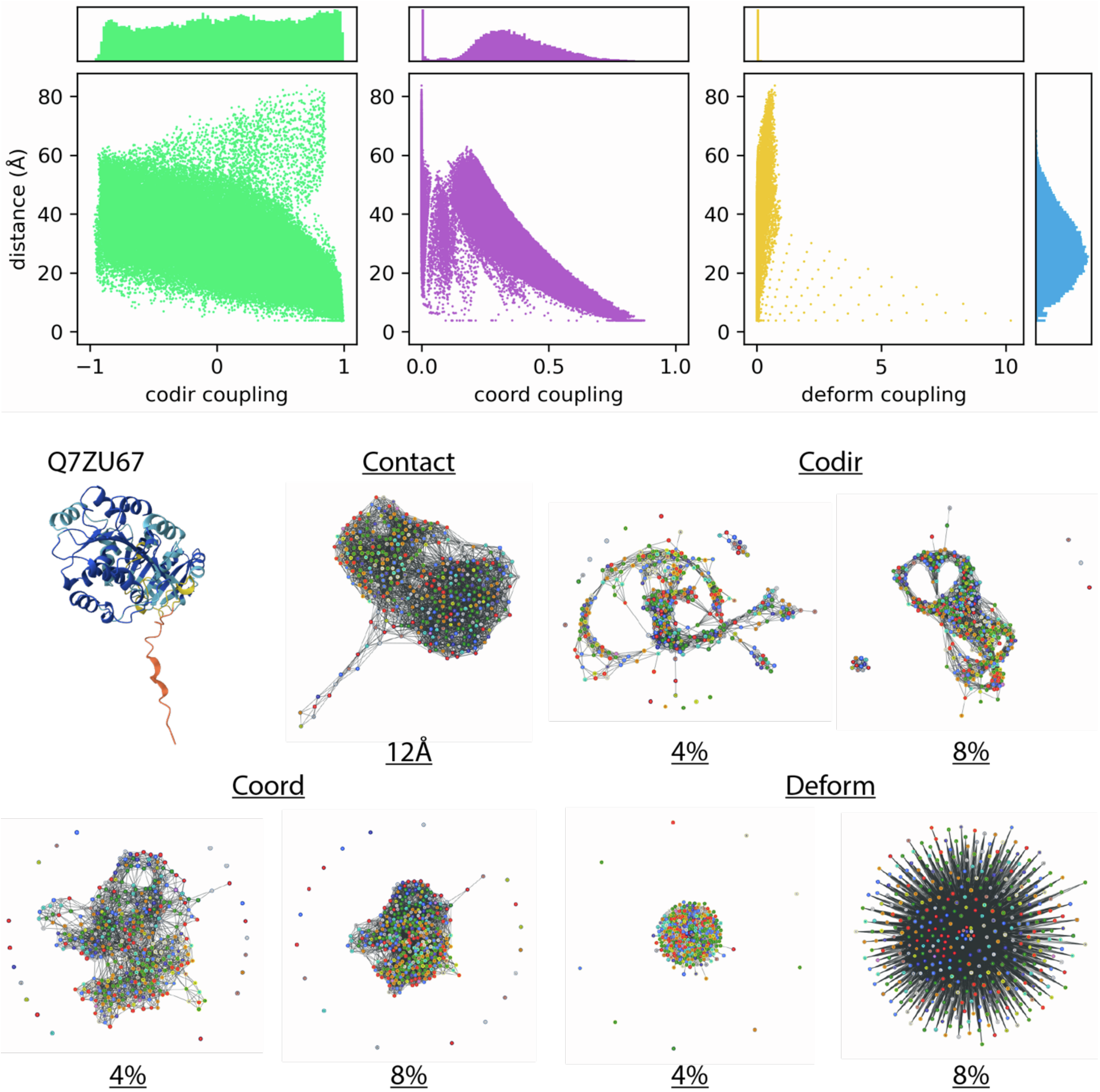

Figure S9. Correlation between coupling values and graph visualization using the scalable force-directed placement (SFDP) layout for UniProt accession Q7ZU67.

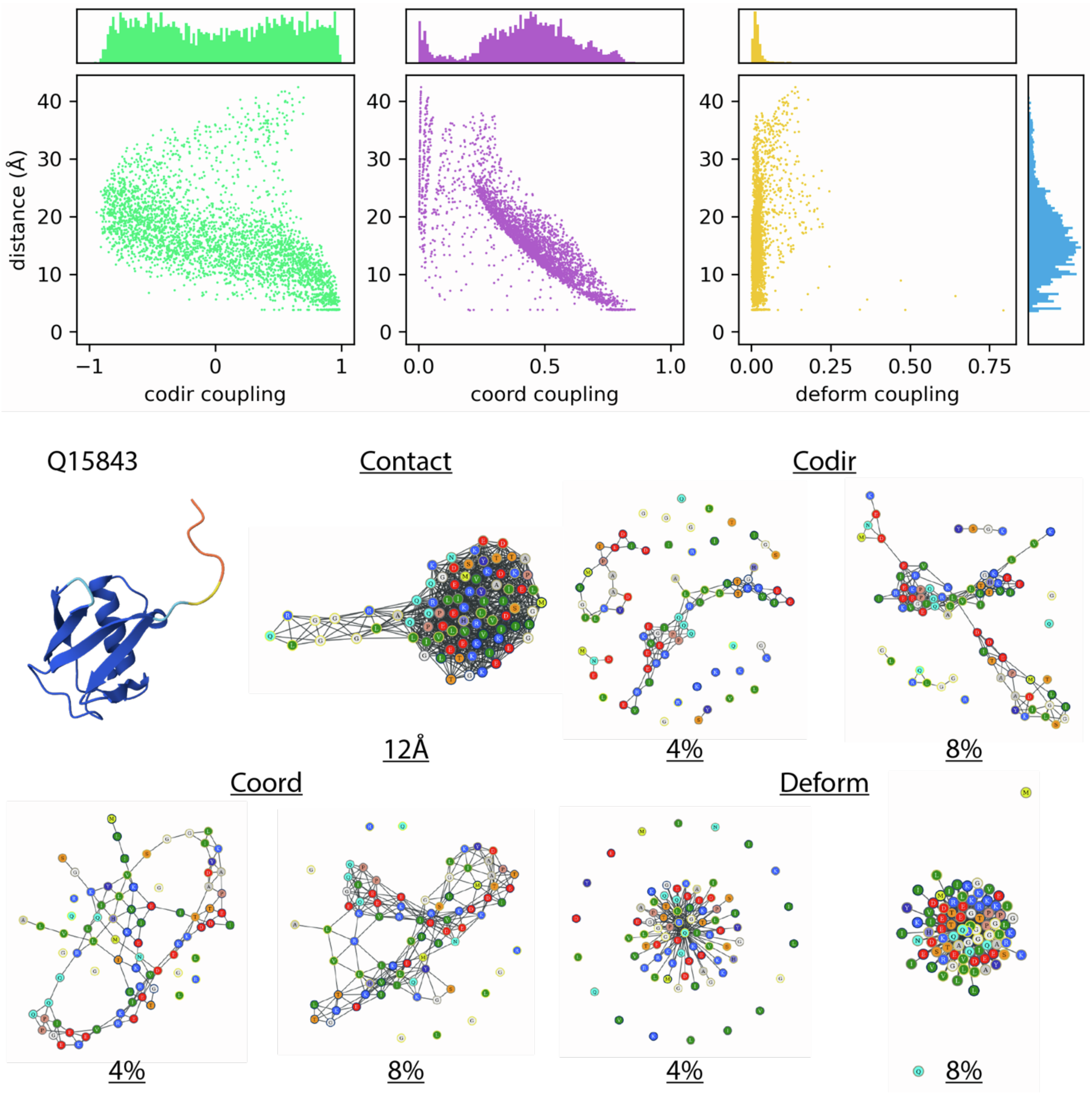

Figure S10. Correlation between coupling values and graph visualization using the scalable force-directed placement (SFDP) layout for UniProt accession Q15843.

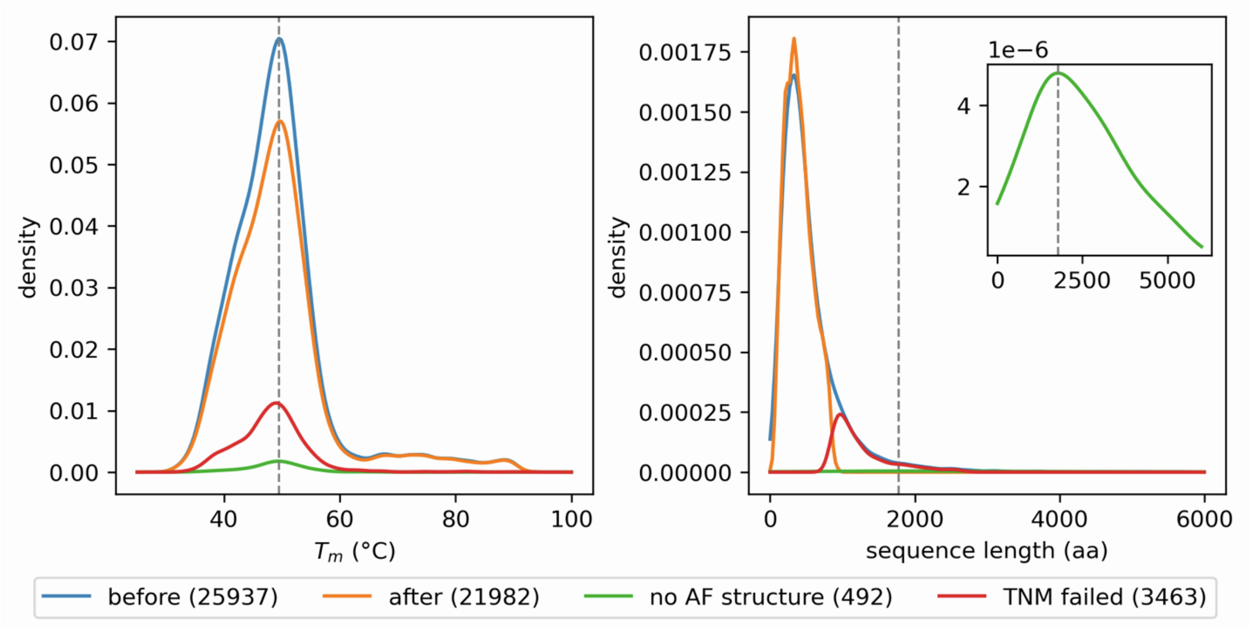

Figure S11. The melting temperature and sequence length distribution for (1) the original DeepSTABp–lysate dataset, (2) the final curated dataset, (3) proteins lacking AlphaFold structures, and (4) proteins for which TNM failed. Values are scaled so that the area under “before” is equal to 1.

Figure S12-S18 shows the performance of all models trained on the preliminary dataset of 896 entries and evaluated on the test set. Models that were not trained are left blank. The backbone-only and contact-only baseline models using the S1 extractor are marked by dashed lines.

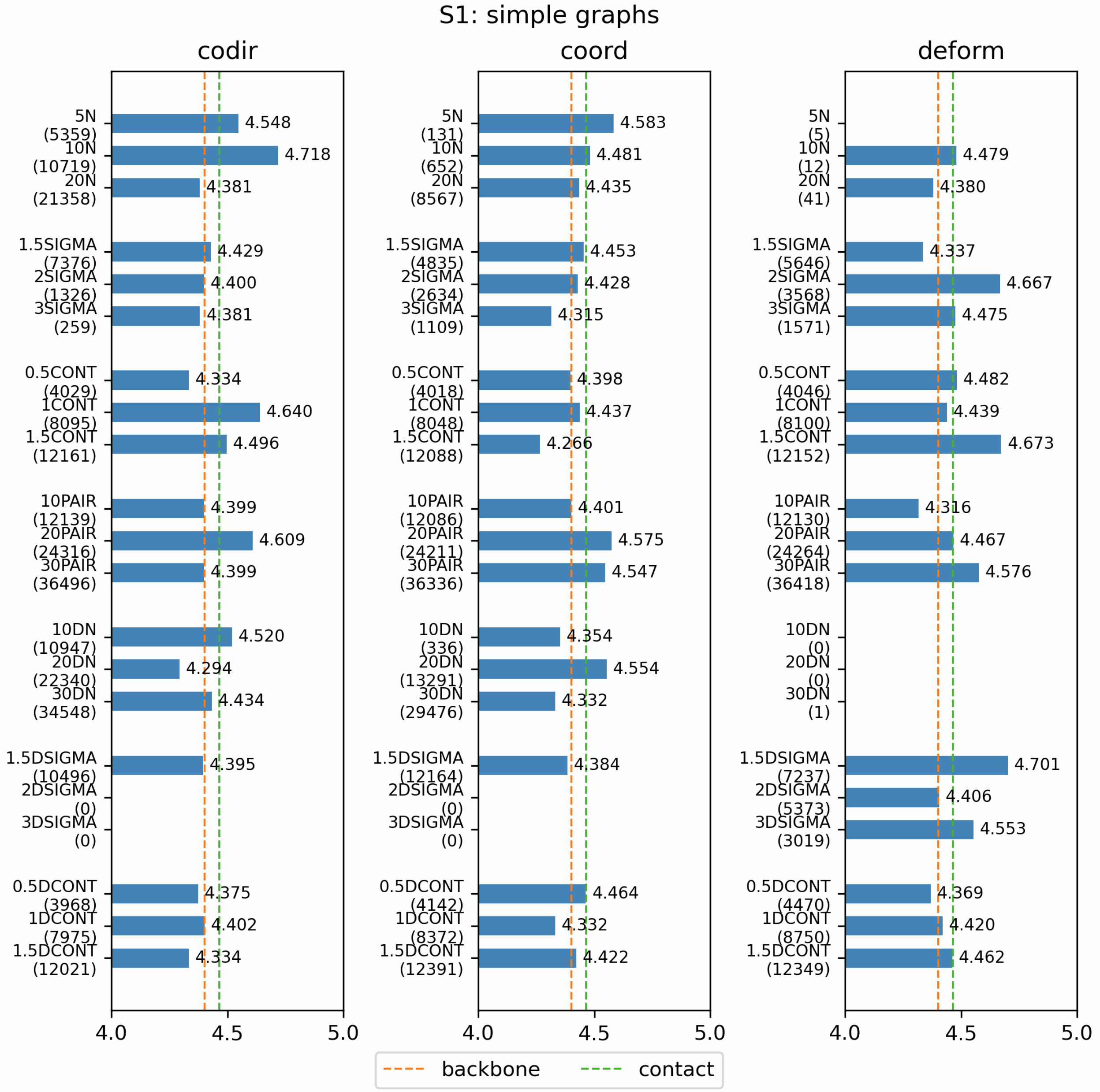

Figure S12. Performance of all models with the S1 extractor and trained on the preliminary dataset. These models are trained using simple graph representations of the edge type specified. The numbers in parentheses show the average number of edges per protein in the dataset.

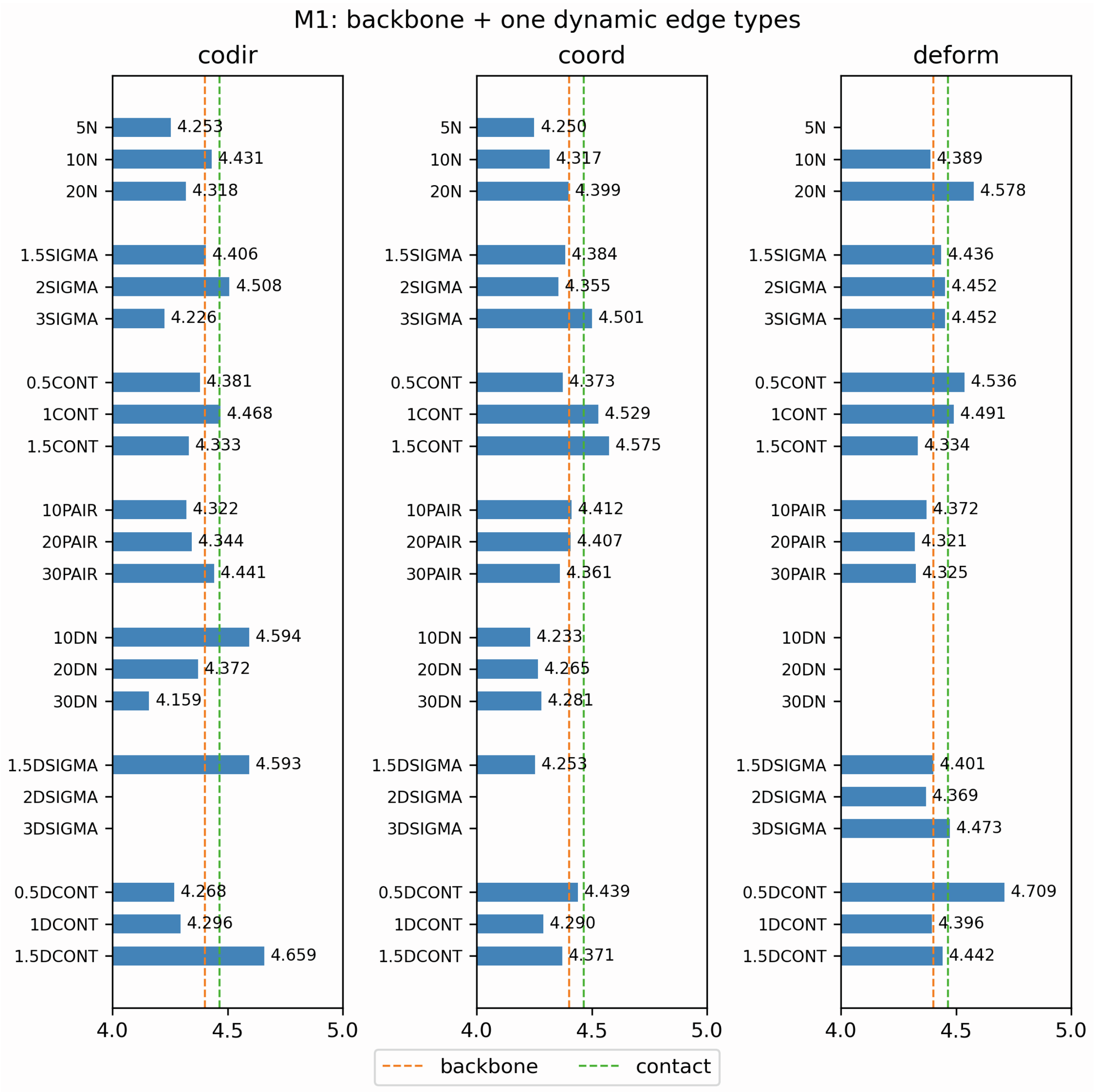

Figure S13. Performance of models with the M1 extractor and trained on the preliminary dataset. These models are trained using multigraph representations with backbone edges and the dynamic edge type specified.

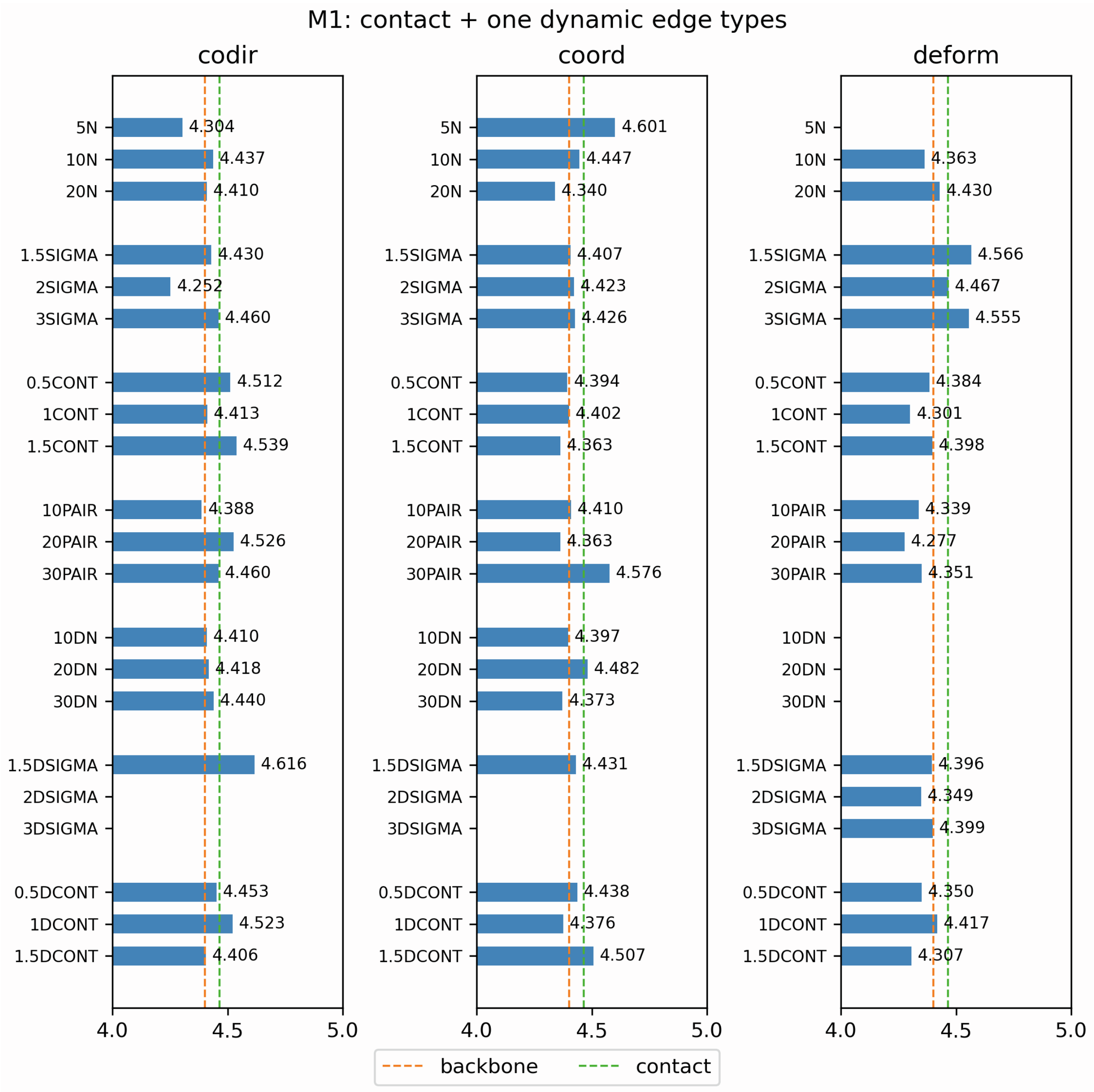

Figure S14. Performance of models with the M1 extractor and trained on the preliminary dataset. These models are trained using multigraph representations with contact edges and the dynamic edge type specified.

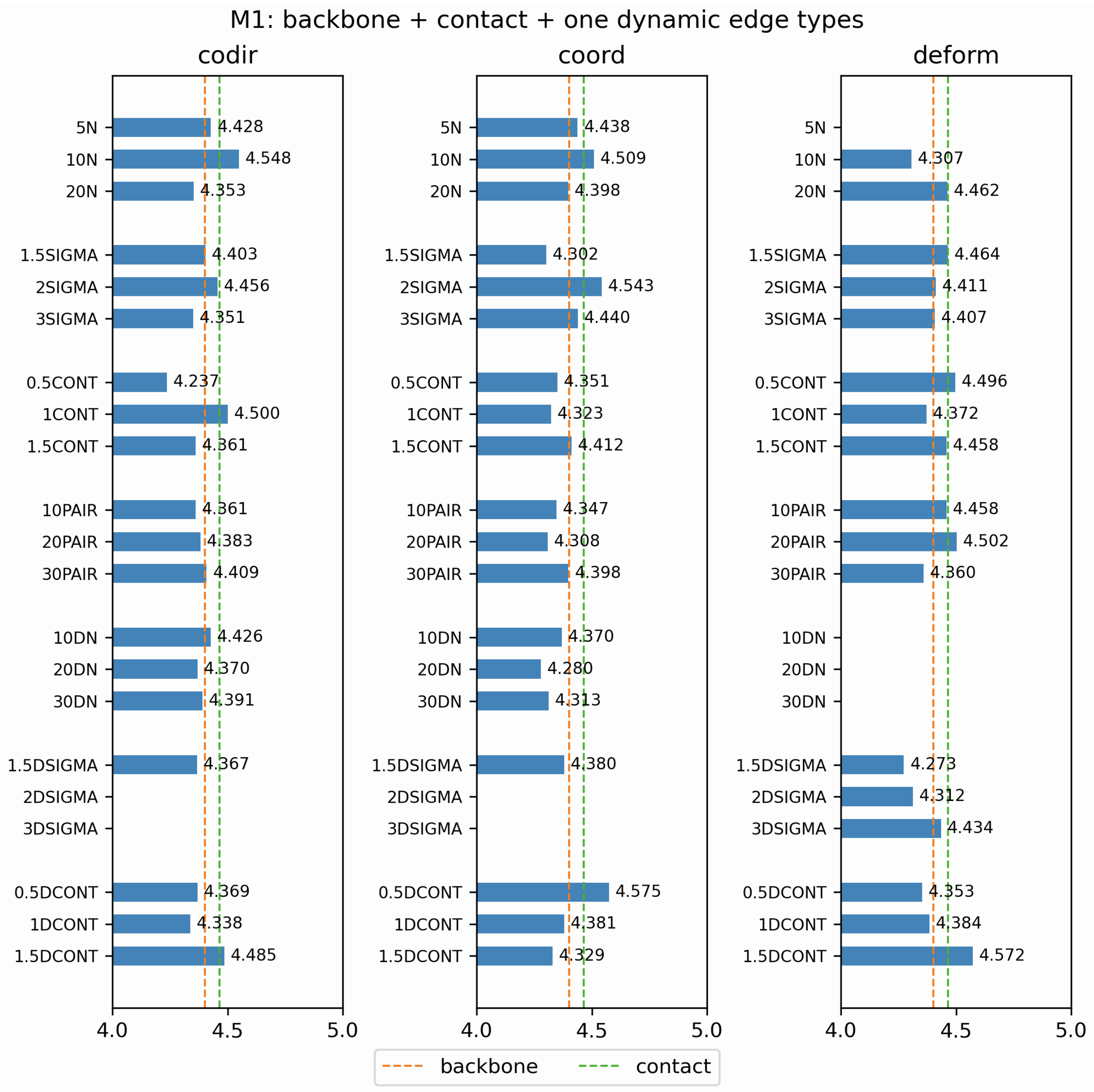

Figure S15. Performance of models with the M1 extractor and trained on the preliminary dataset. These models are trained using multigraph representations with backbone edges, contact edges, and the dynamic edge type specified.

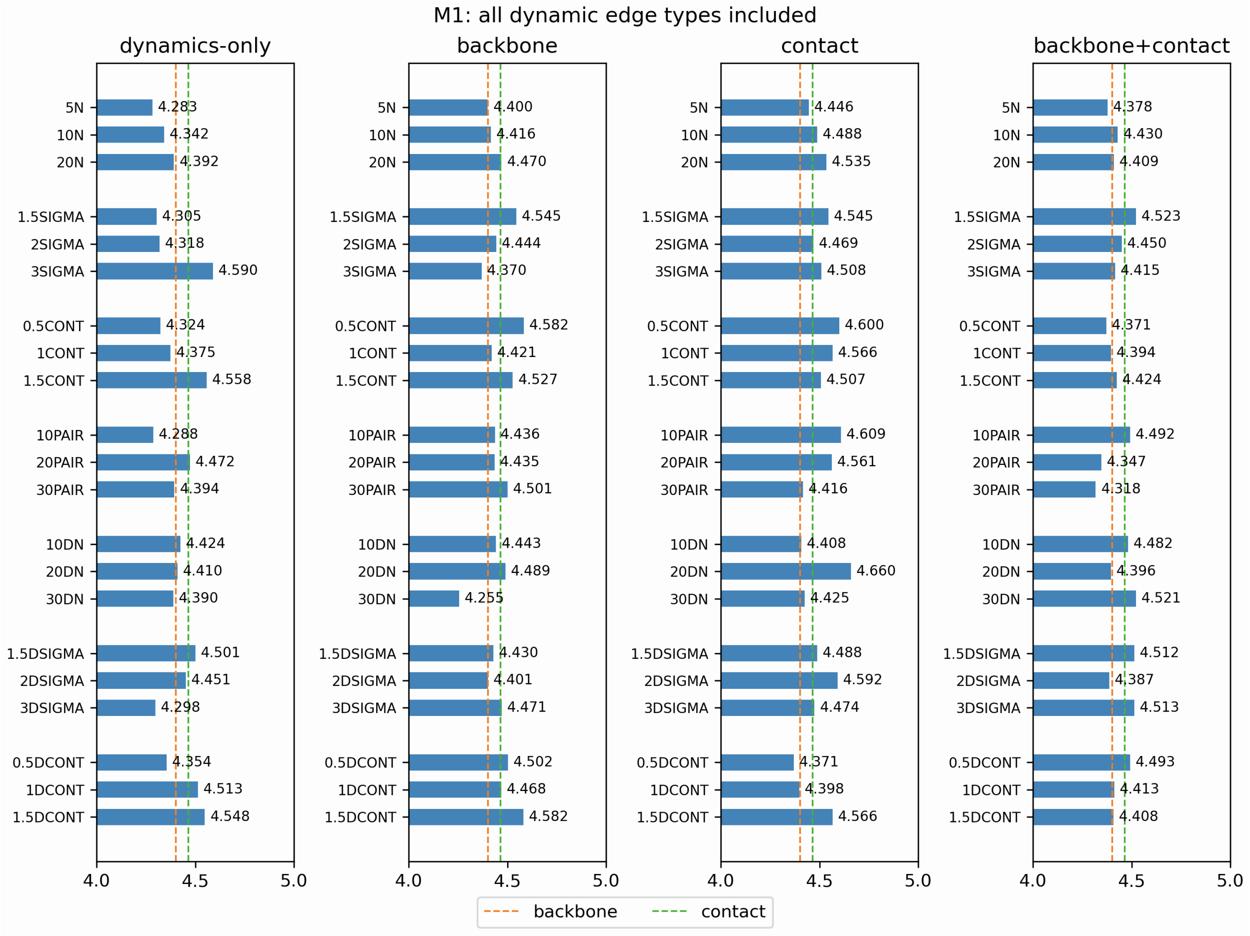

Figure S16. Performance of models with the M1 extractor and trained on the preliminary dataset. These models are trained using multigraph representations with all dynamic edge types included, plus the combination of non-dynamics edge types specified.

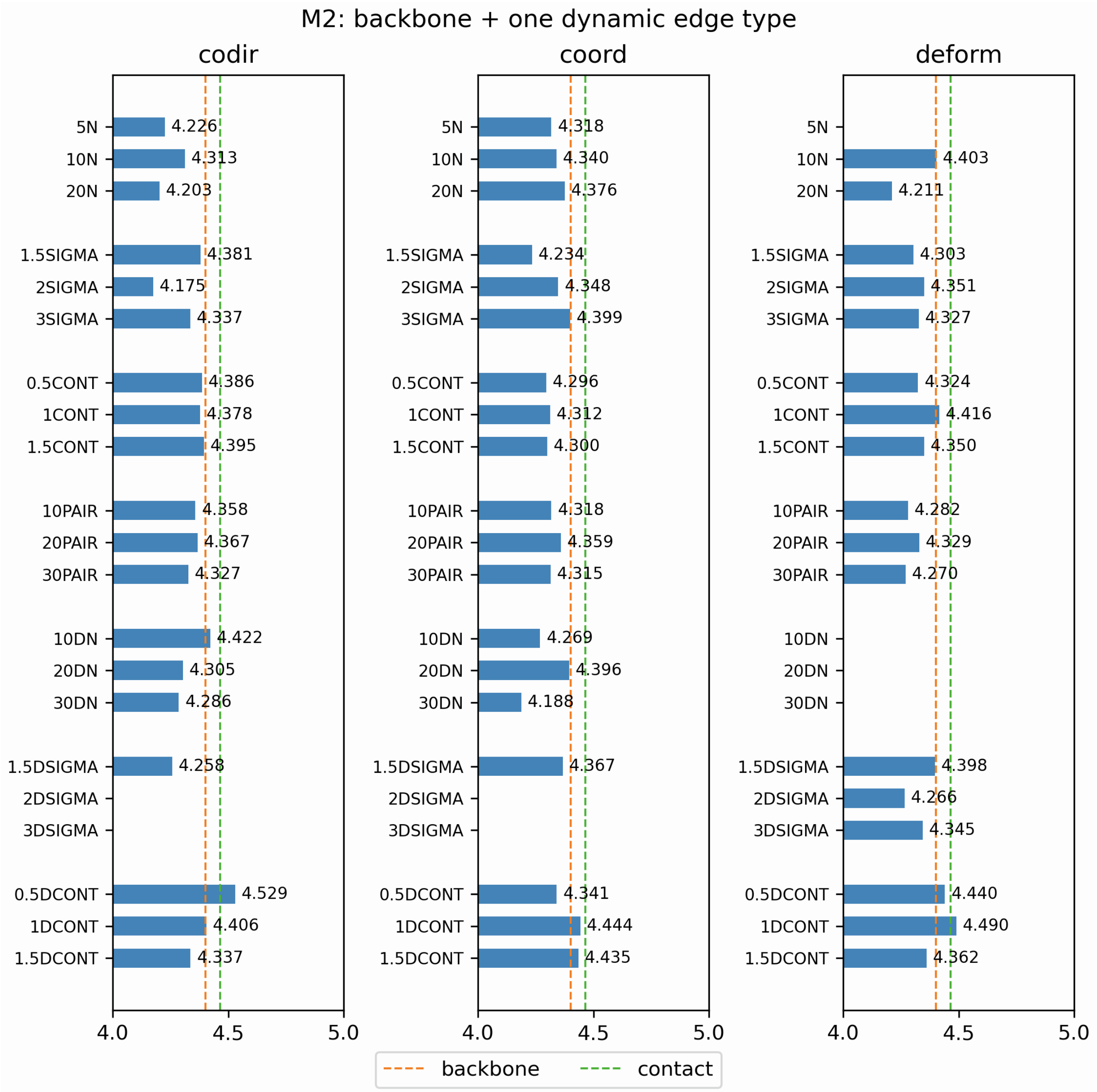

Figure S17. Performance of models with the M2 extractor and trained on the preliminary dataset. These models are trained using multigraph representations with backbone edges and the dynamic edge type specified.

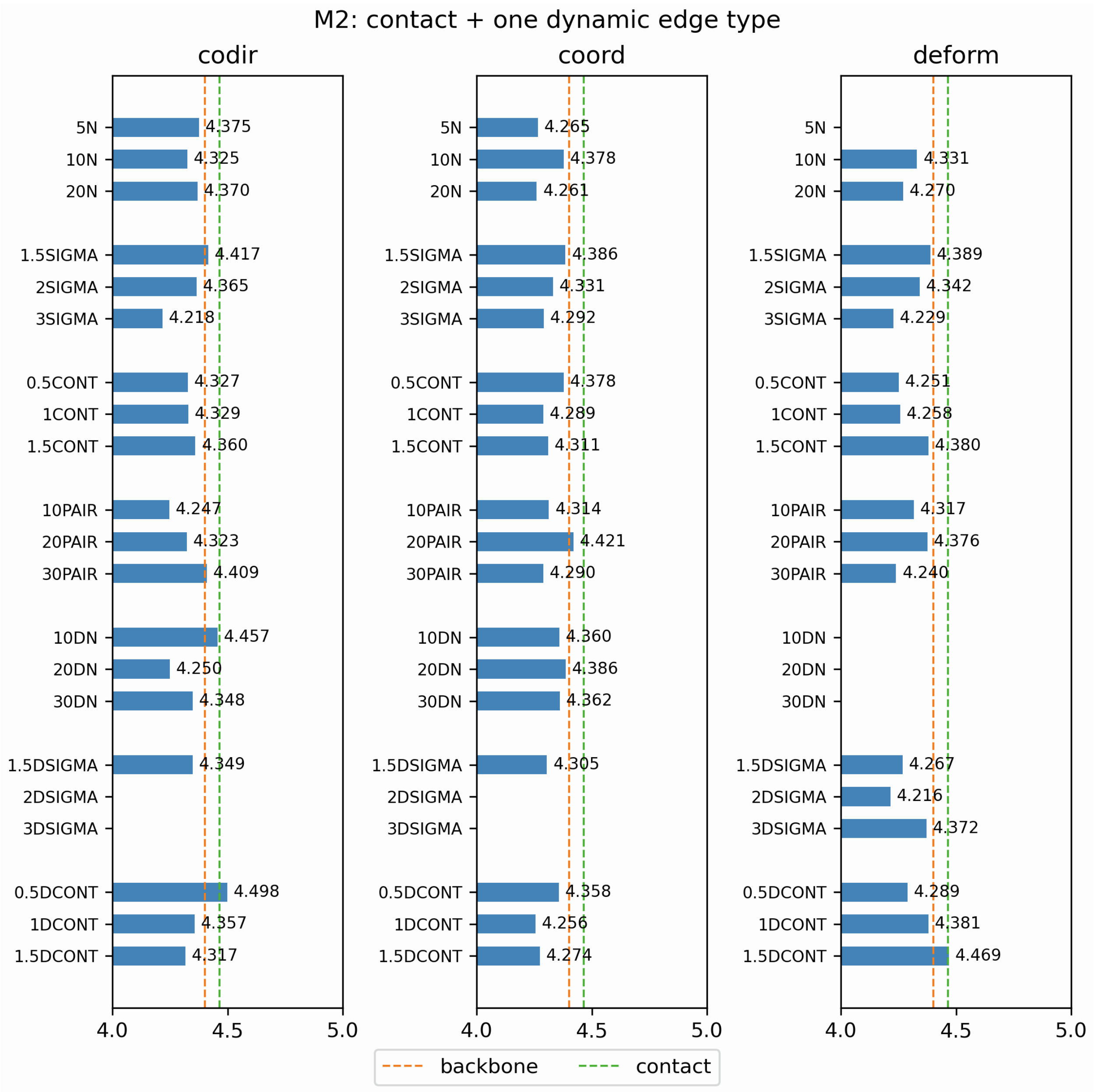

Figure S18. Performance of models with the M2 extractor and trained on the preliminary dataset. These models are trained using multigraph representations with contact edges and the dynamic edge type specified.

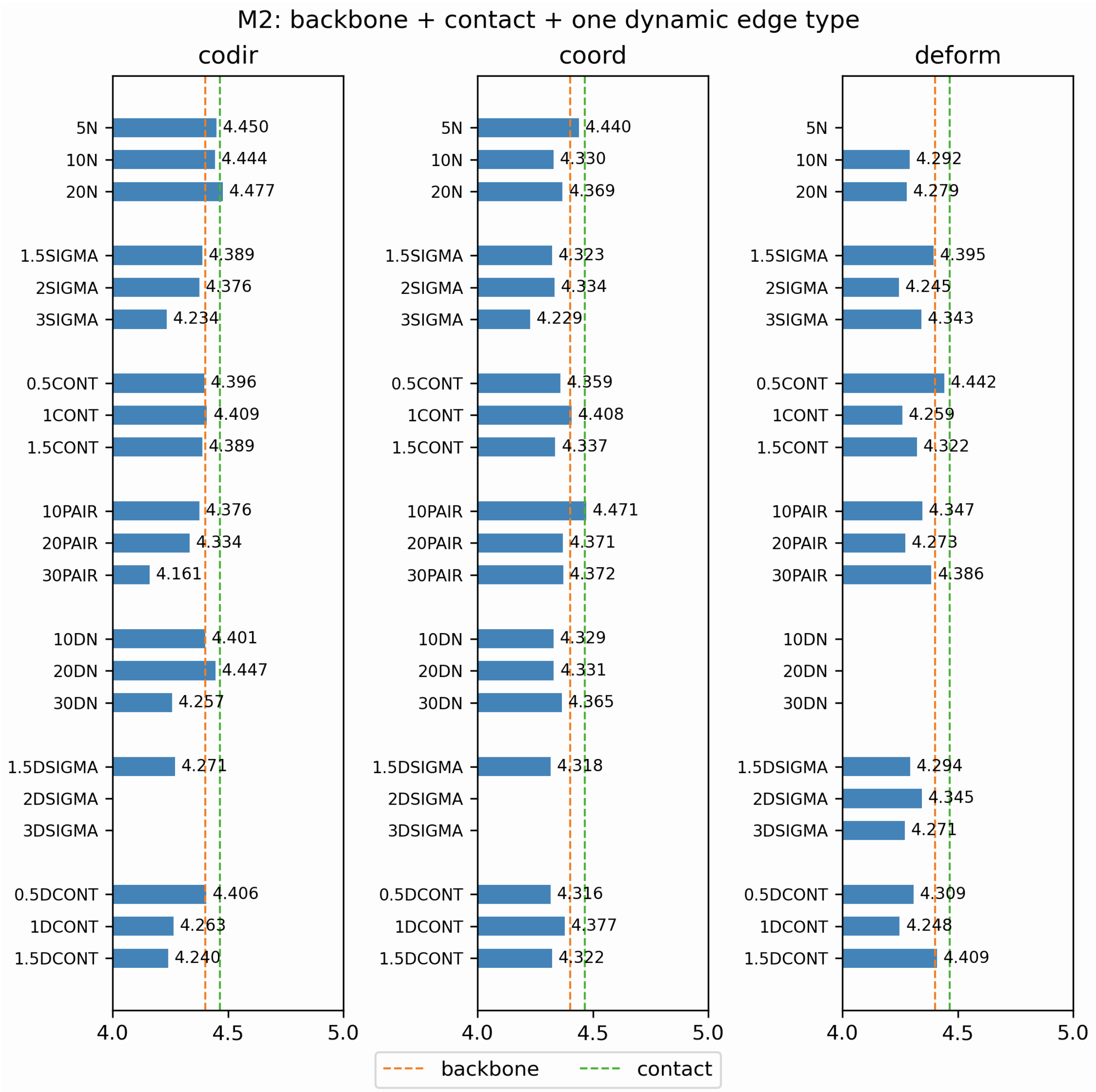

Figure S19. Performance of models with the M2 extractor and trained on the preliminary dataset. These models are trained using multigraph representations with backbone edges, contact edges, and the dynamic edge type specified.

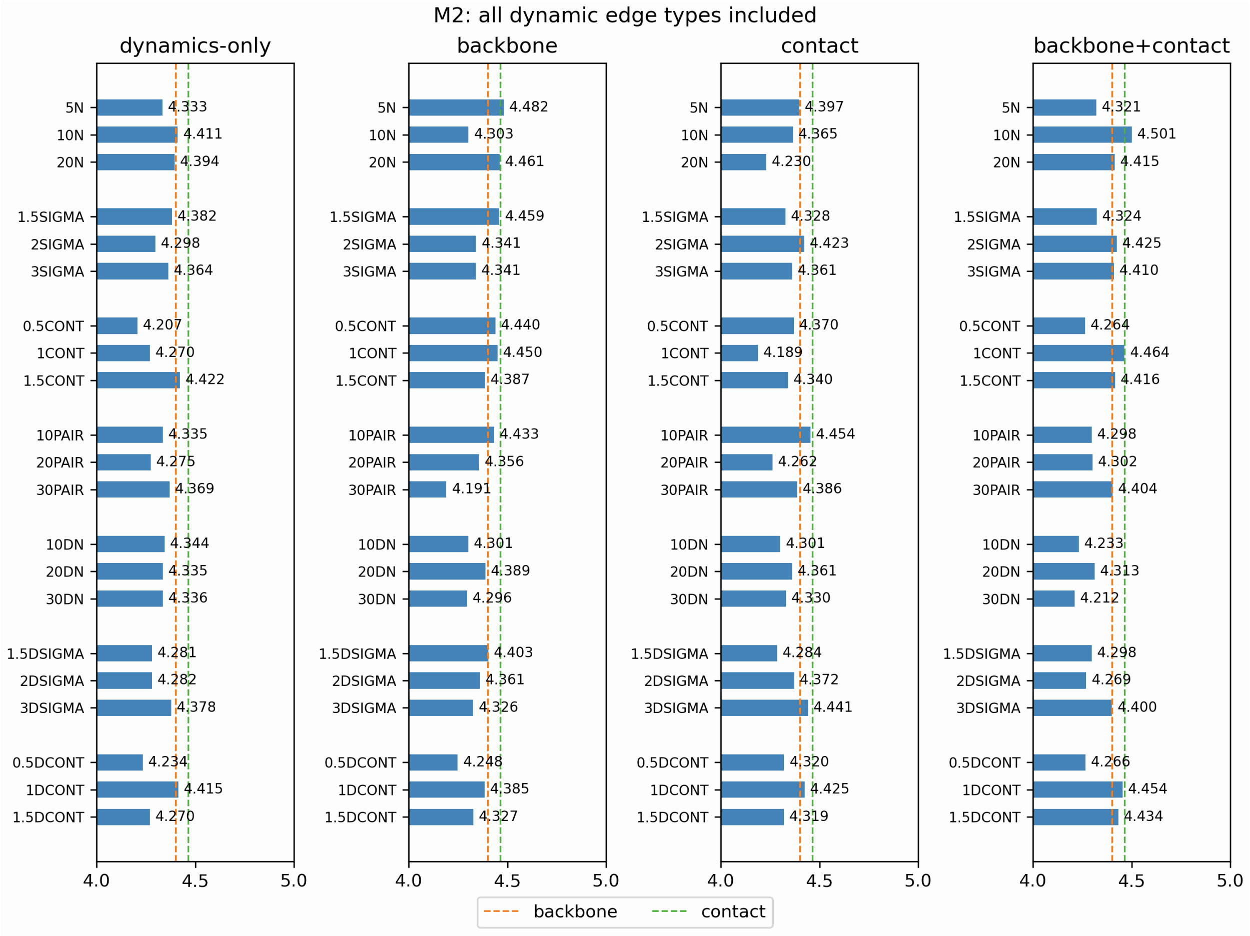

Figure S20. Performance of models with the M2 extractor and trained on the preliminary dataset. These models are trained using multigraph representations with all dynamic edge types included, plus the combination of non-dynamics edge types specified.

Figure S21-S29 shows the performance of all models trained on the full training dataset of 19,938 entries and evaluated on the test set. Models that were not trained are left blank. The backbone-only and contact-only baseline models using the S1 extractor are marked by dashed lines.

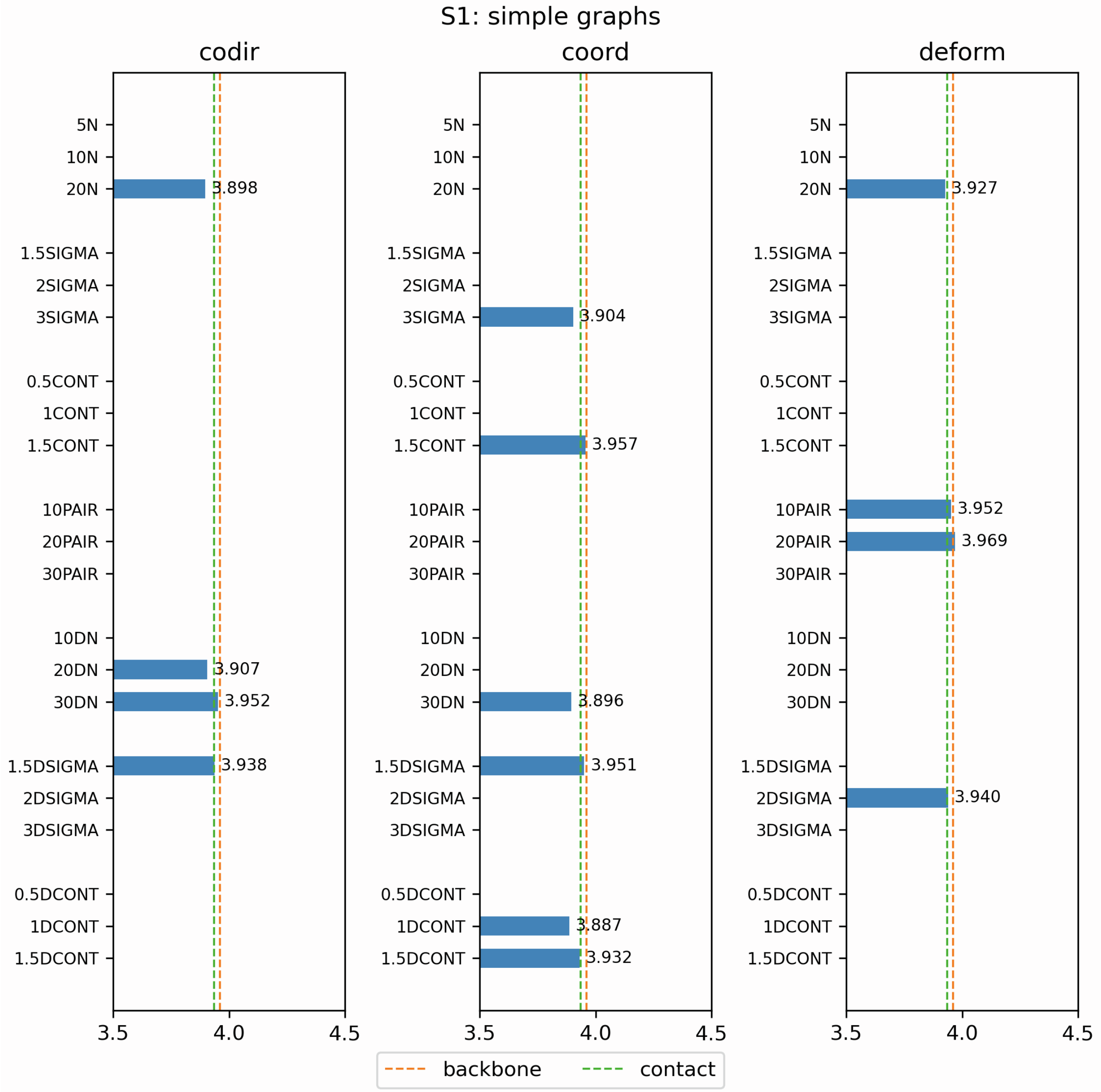

Figure S21. Performance of all models with the S1 extractor and trained on the full training dataset. These models are trained using simple graph representations of the edge type specified.

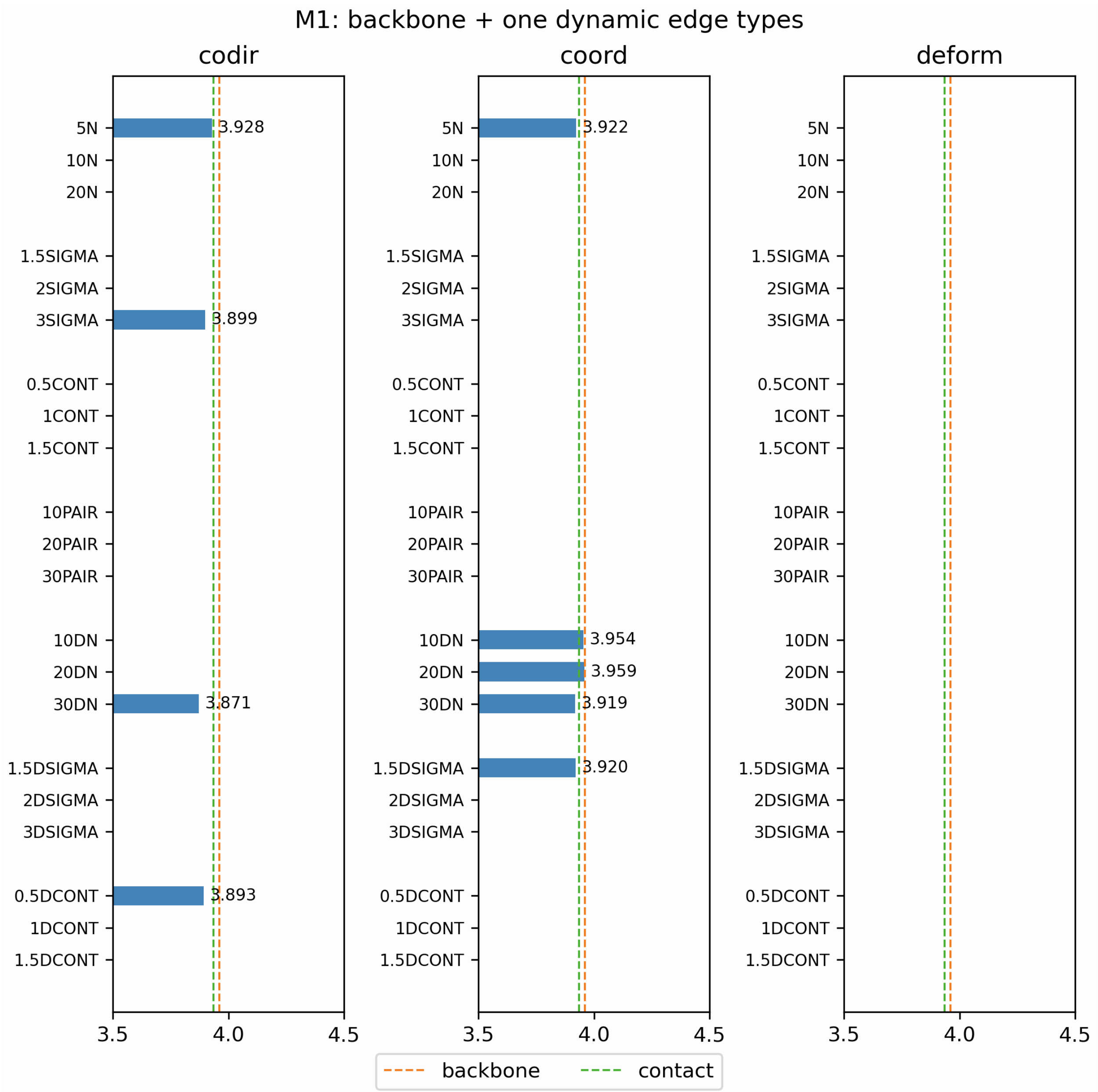

Figure S22. Performance of models with the M1 extractor and trained on the full training dataset. These models are trained using multigraph representations with backbone edges and the dynamic edge type specified.

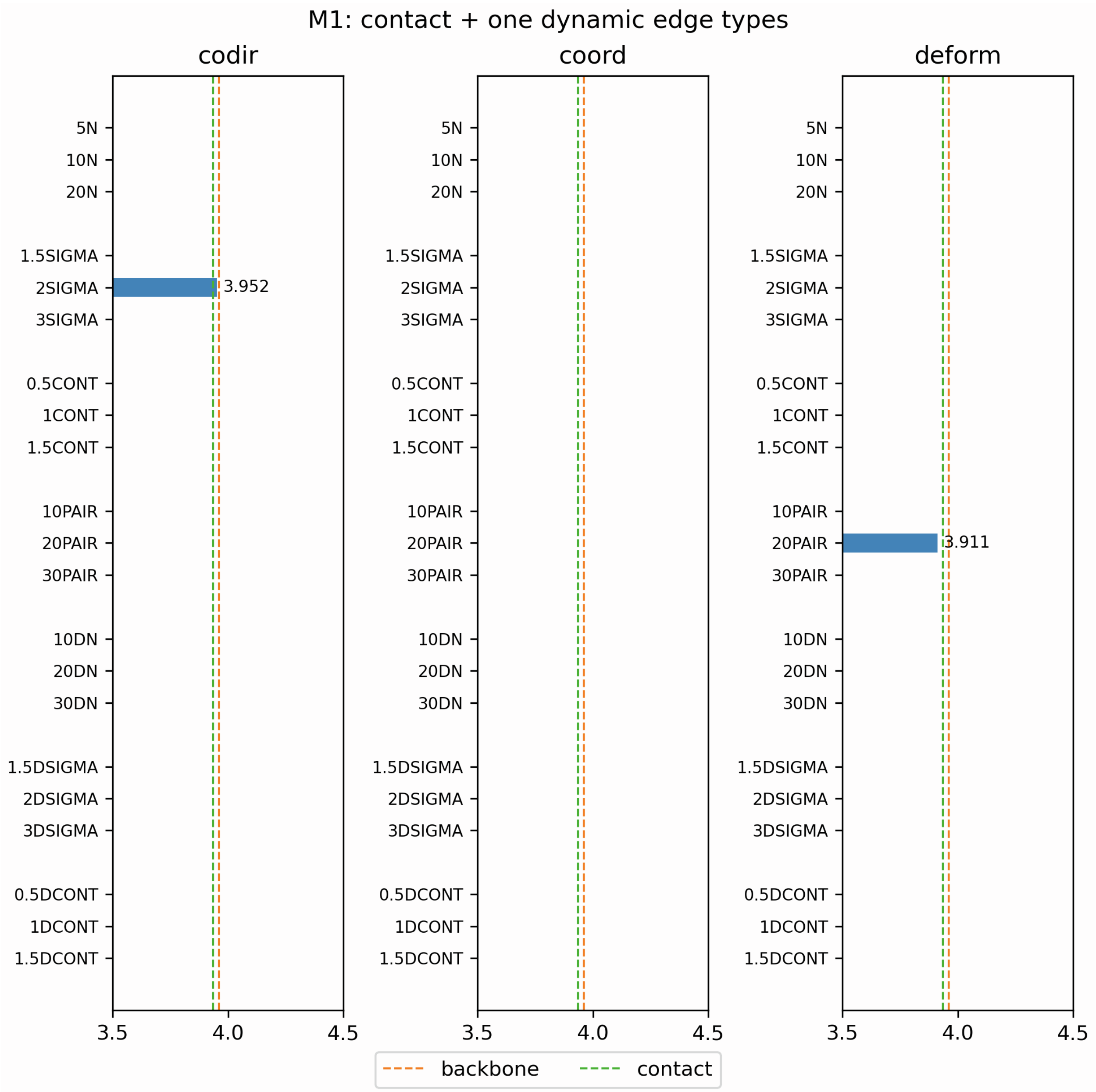

Figure S23. Performance of models with the M1 extractor and trained on the full training dataset. These models are trained using multigraph representations with contact edges and the dynamic edge type specified.

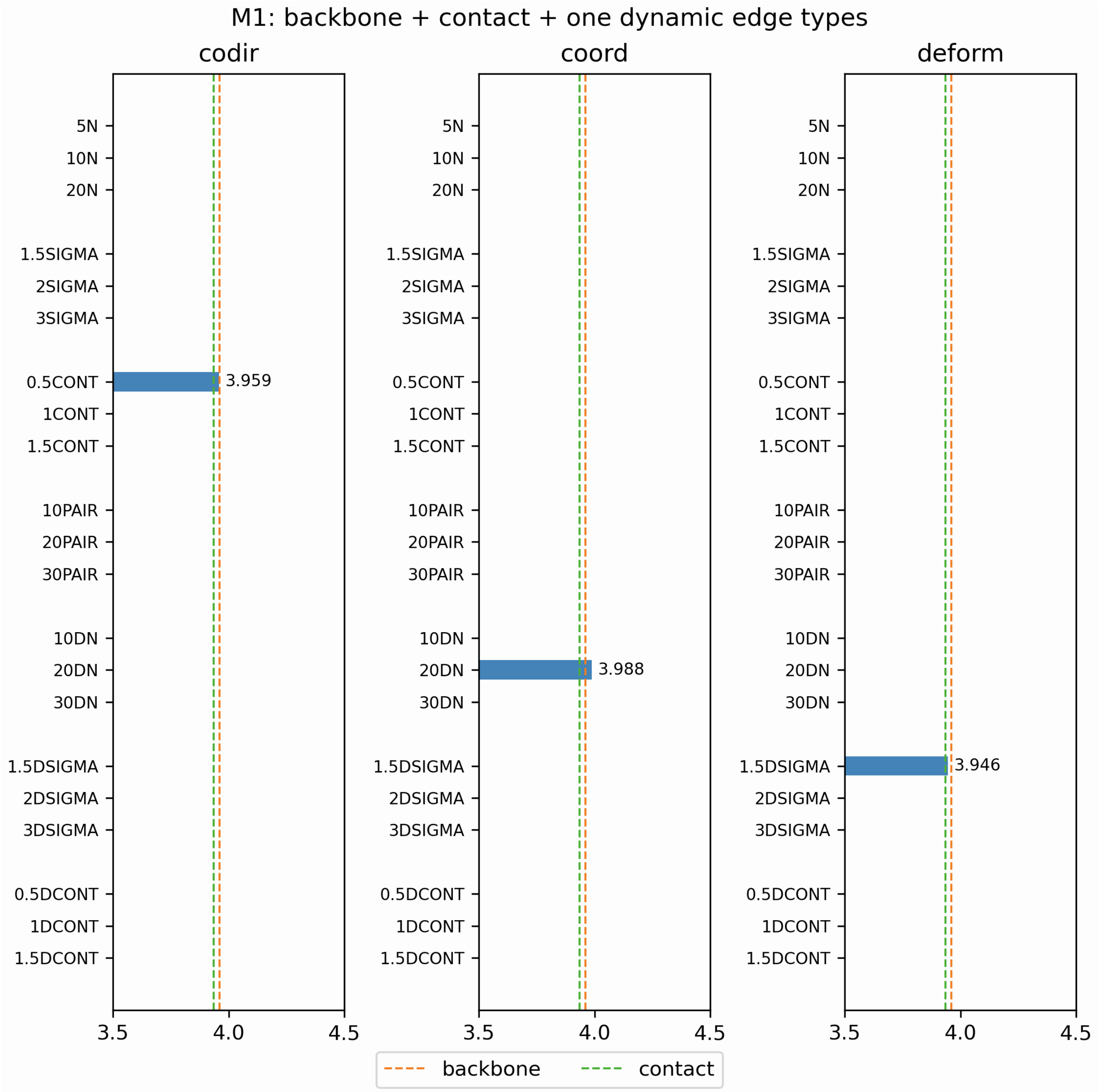

Figure S24. Performance of models with the M1 extractor and trained on the full training dataset. These models are trained using multigraph representations with backbone edges, contact edges, and the dynamic edge type specified.

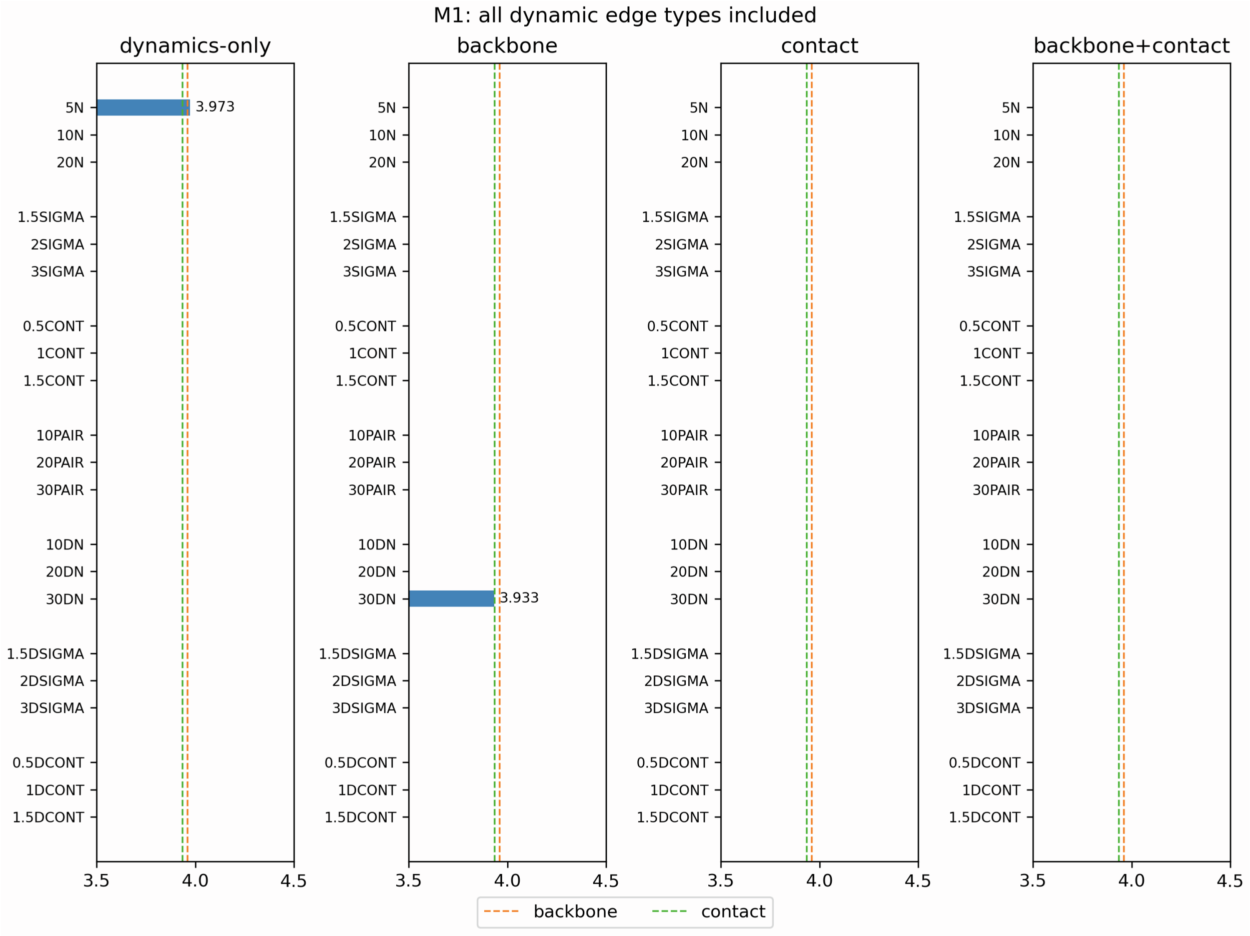

Figure S25. Performance of models with the M1 extractor and trained on the full training dataset. These models are trained using multigraph representations with all dynamic edge types included, plus the combination of non-dynamics edge types specified.

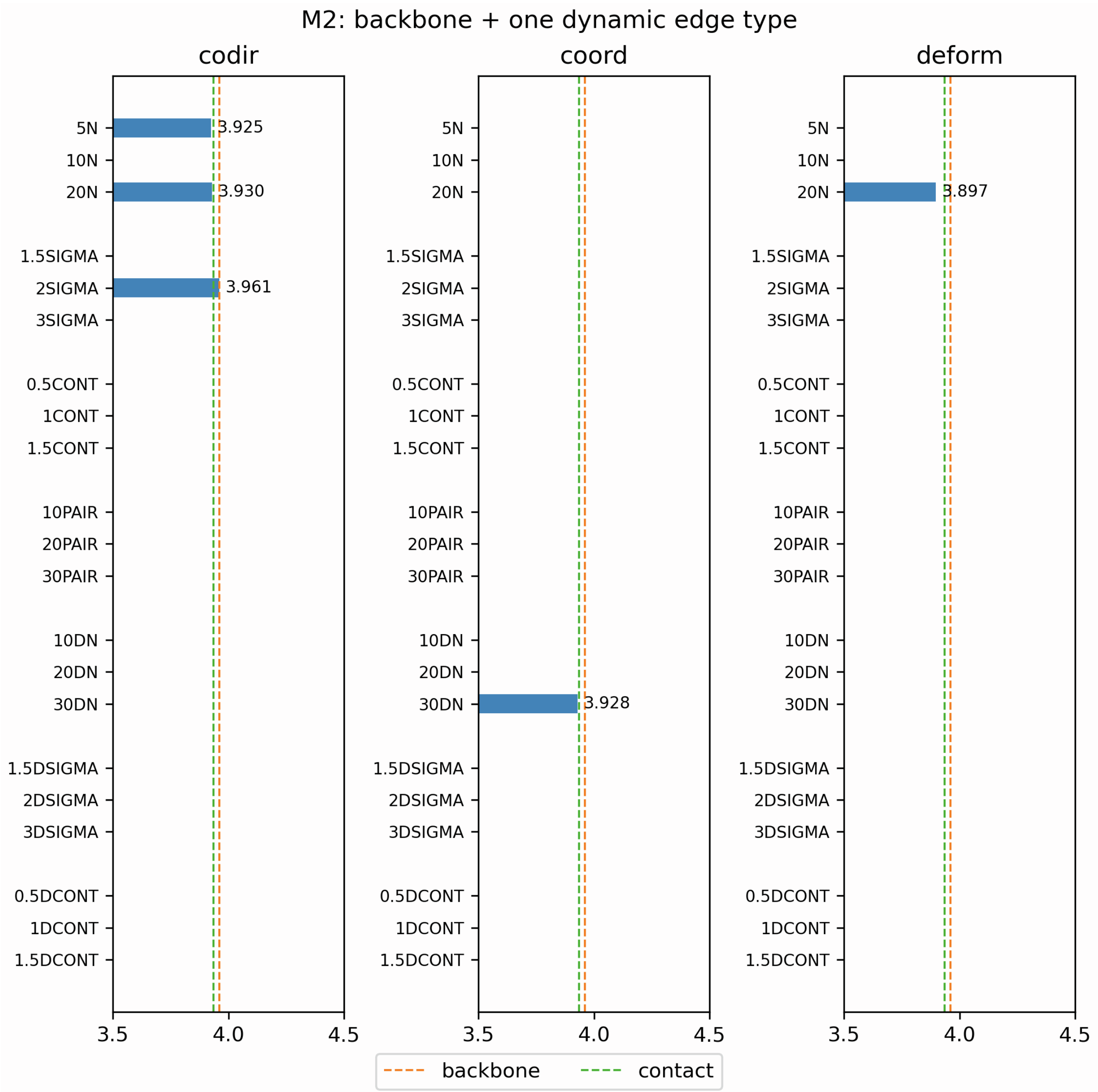

Figure S26. Performance of models with the M2 extractor and trained on the full training dataset. These models are trained using multigraph representations with backbone edges and the dynamic edge type specified.

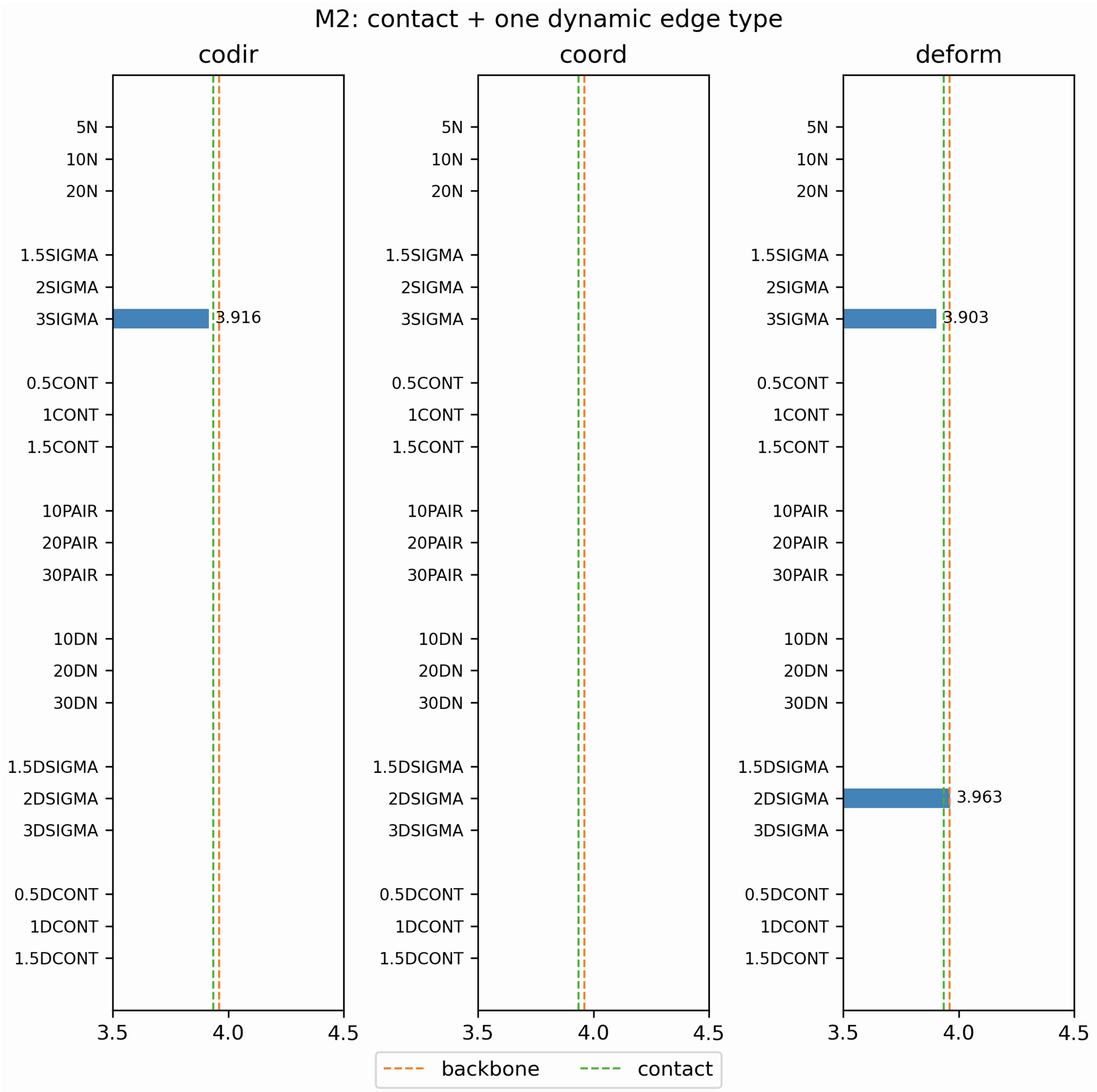

Figure S27. Performance of models with the M2 extractor and trained on the full training dataset. These models are trained using multigraph representations with contact edges and the dynamic edge type specified.

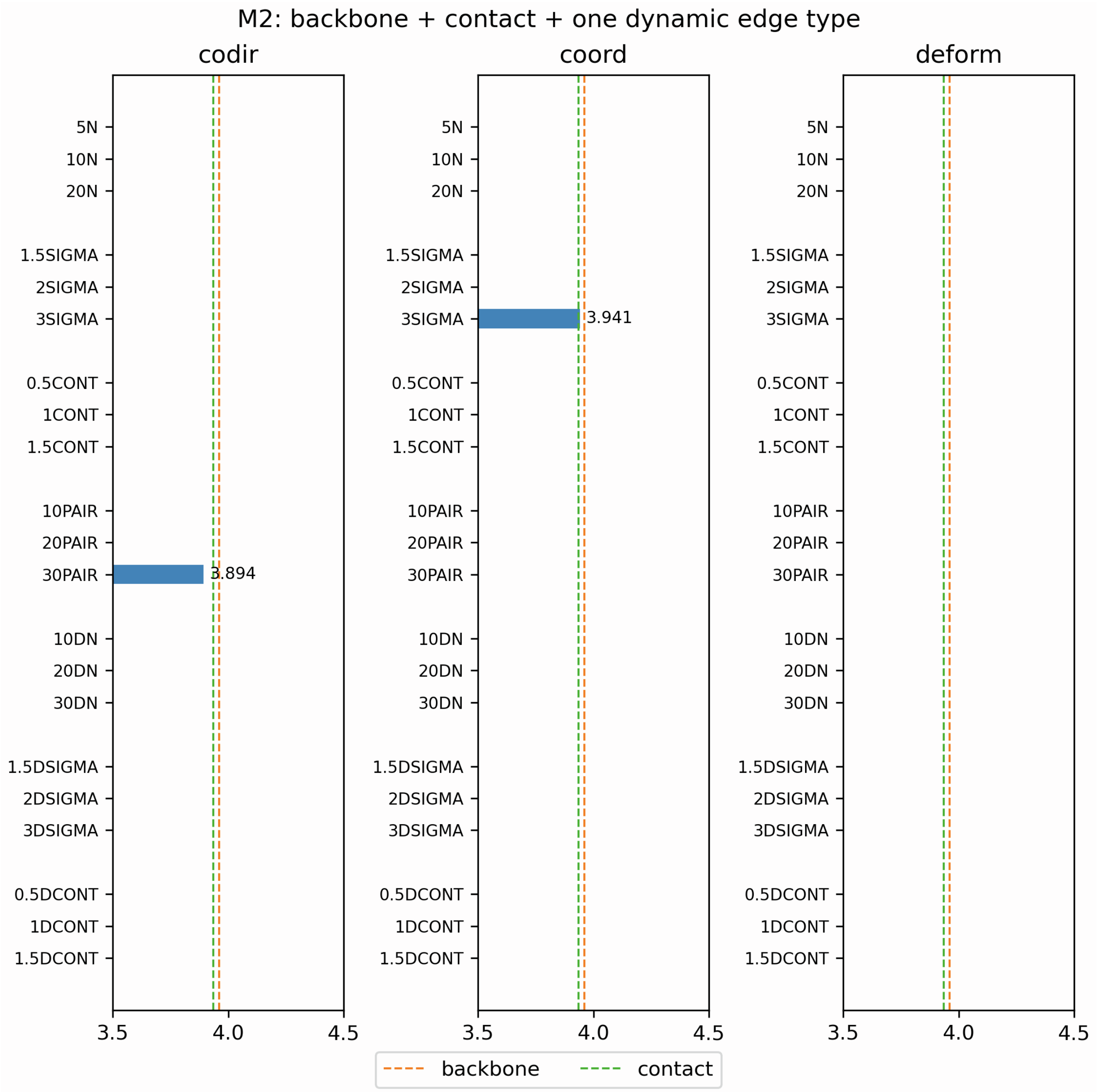

Figure S28. Performance of models with the M2 extractor and trained on the full training dataset. These models are trained using multigraph representations with backbone edges, contact edges, and the dynamic edge type specified.

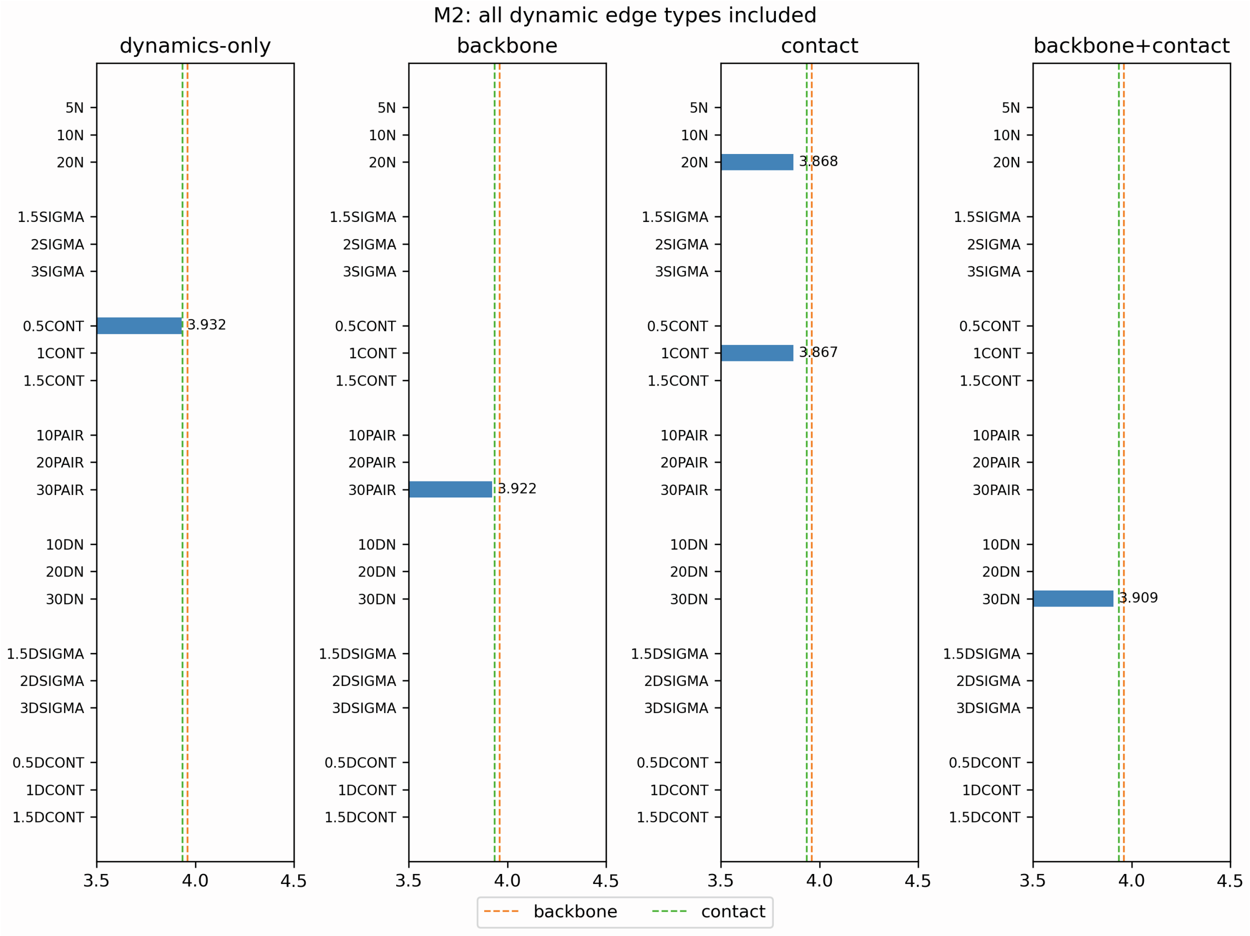

Figure S29. Performance of models with the M2 extractor and trained on the full training dataset. These models are trained using multigraph representations with all dynamic edge types included, plus the combination of non-dynamics edge types specified.

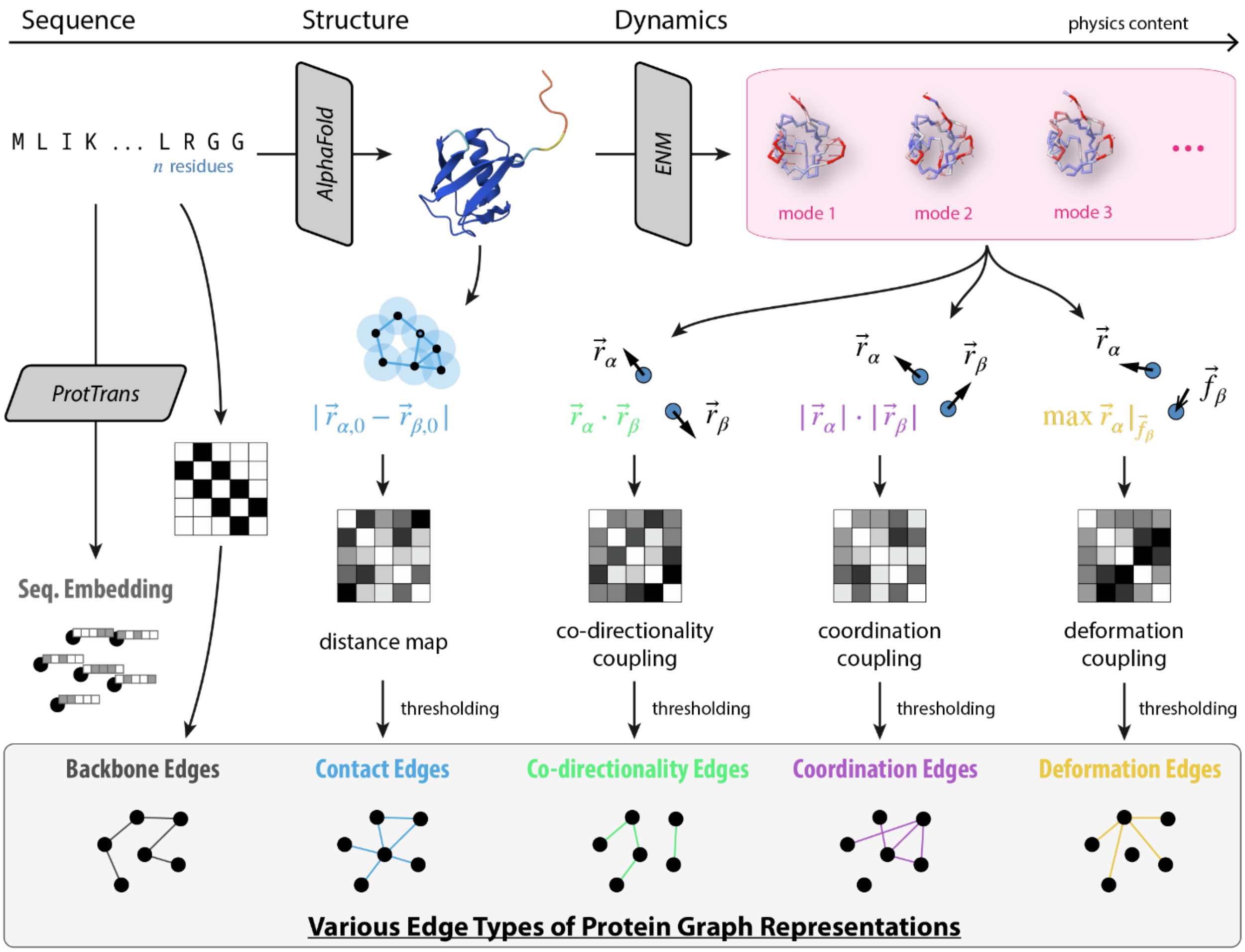

Figure S30. Overview of the proposed graph representations for proteins.

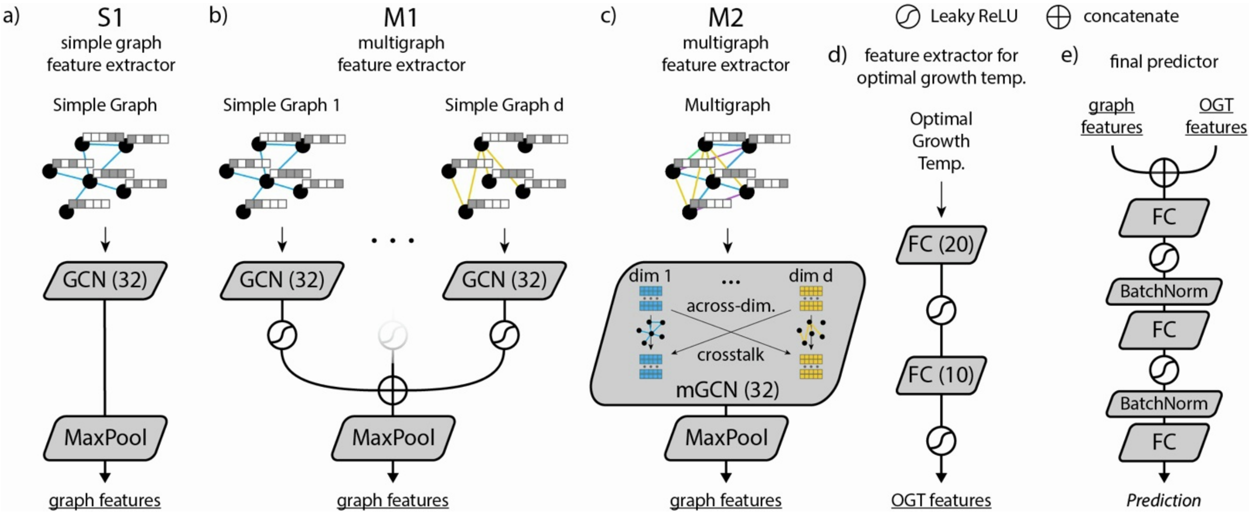

Figure S31. Three types of feature extractors were used in this work to process the graph representation and extract the graph-level feature. (a) The S1 feature extractor is used to process simple graph representations. (b) The M1 extractor uses a GCN layer to deal with each edge type, then concatenates and max-pools the node features to obtain the graph-level feature. (c) The M2 extractor uses an mGCN aggregation layer, followed by a max-pooling layer, to produce the graph-level feature. The mGCN module aggregates information across multiple graph types through learned across-dimension crosstalk, enabling information exchange between graph dimensions. (d) The feature extractor for optimal growth temperature of the species uses a series of neural layers and activations to produce the OGT feature. (e) The graph-level feature and the OGT feature are concatenated, then processed by a final predictor to produce the final prediction.
